# Genetic complexity of miscanthus cell wall composition and biomass quality for biofuels

**DOI:** 10.1186/s12864-017-3802-7

**Published:** 2017-05-25

**Authors:** Tim van der Weijde, Claire L. Alvim Kamei, Edouard I. Severing, Andres F. Torres, Leonardo D. Gomez, Oene Dolstra, Chris A. Maliepaard, Simon J. McQueen-Mason, Richard G. F. Visser, Luisa M. Trindade

**Affiliations:** 1 Wageningen UR Plant Breeding, Wageningen University and Research, PO Box 386, 6700 AJ Wageningen, Netherlands; 2 Graduate School Experimental Plant Sciences, Wageningen University, 6708 PB Wageningen, Netherlands; 3 Present address: Research, Barenbrug Holland B.V, Duitsekampweg 60, 6748 ZB Wolfheze, Netherlands; 4 Present address: Department of Comparative Development and Genetics, Max Planck Institute for Plant Breeding Research, Carl-von-Linné-Weg 10, 50829 Cologne, Germany; 5 Present address: Department of Plant Developmental Biology, Max Planck Institute for Plant Breeding Research, Carl-von-Linné-Weg 10, 50829 Cologne, Germany; 6 Center for Novel Agricultural Products, University of York, YO10 5 DD York, UK

**Keywords:** Miscanthus, Biofuel, Quantitative trait loci (QTL), Genetic map, Yield, Biomass quality, Cell wall composition, Saccharification efficiency, Conversion efficiency

## Abstract

**Background:**

*Miscanthus sinensis* is a high yielding perennial grass species with great potential as a bioenergy feedstock. One of the challenges that currently impedes commercial cellulosic biofuel production is the technical difficulty to efficiently convert lignocellulosic biomass into biofuel. The development of feedstocks with better biomass quality will improve conversion efficiency and the sustainability of the value-chain. Progress in the genetic improvement of biomass quality may be substantially expedited by the development of genetic markers associated to quality traits, which can be used in a marker-assisted selection program.

**Results:**

To this end, a mapping population was developed by crossing two parents of contrasting cell wall composition. The performance of 182 F1 offspring individuals along with the parents was evaluated in a field trial with a randomized block design with three replicates. Plants were phenotyped for cell wall composition and conversion efficiency characters in the second and third growth season after establishment. A new SNP-based genetic map for *M. sinensis* was built using a genotyping-by-sequencing (GBS) approach, which resulted in 464 short-sequence uniparental markers that formed 16 linkage groups in the male map and 17 linkage groups in the female map. A total of 86 QTLs for a variety of biomass quality characteristics were identified, 20 of which were detected in both growth seasons. Twenty QTLs were directly associated to different conversion efficiency characters. Marker sequences were aligned to the sorghum reference genome to facilitate cross-species comparisons. Analyses revealed that for some traits previously identified QTLs in sorghum occurred in homologous regions on the same chromosome.

**Conclusion:**

In this work we report for the first time the genetic mapping of cell wall composition and bioconversion traits in the bioenergy crop miscanthus. These results are a first step towards the development of marker-assisted selection programs in miscanthus to improve biomass quality and facilitate its use as feedstock for biofuel production.

**Electronic supplementary material:**

The online version of this article (doi:10.1186/s12864-017-3802-7) contains supplementary material, which is available to authorized users.

## Background

Miscanthus is a perennial C4 grass capable of producing high biomass yields in temperate climates [1]. It is a crop characterized by high resource-use efficiency owing to its early spring emergence and long vegetative phase, as well as its rhizomatous growing habit, which allows the recycling of nutrients between growing seasons [2–4]. These characteristics make miscanthus an interesting lignocellulose feedstock for the production of cellulosic biofuels [5].

So far, *M. × giganteus* is the only species of the genus *Miscanthus* that is commercially exploited for biomass production [6, 7]. *M. × giganteus* (*2n = 3x = 57*) is derived from a natural cross between the diploid *M. sinensis* (*2n = 2 × = 38*) and the Japanese allotetraploid species *M. ogiformis* (*2n = 4 × = 76*), which is often erroneously referred to as tetraploid *M. sacchariflorus* [8, 9]. Its success is mainly due to its high productivity. In a quantitative review of biomass yields of *M. × giganteus* across 100 diverse field trial locations, the average dry matter yield was 22 t ha^−1^ yr^−1^ [10]. However, the genetic variation in this triploid clone is extremely limited due to its sterility, which poses risks upon large-scale cultivation and significantly limits further progress through breeding [6, 9, 11–13]. In contrast, great and largely untapped genetic diversity is harboured within and among natural populations of *M. sacchariflorus* and *M. sinensis*, which have adapted to a wide range of geographical conditions [6, 13].

One of the key challenges that currently impedes the wide-scale commercialization of cellulosic ethanol production resides is our inability to efficiently deconstruct plant lignocellulose into fermentable sugars. The development of feedstocks with improved biomass quality is envisioned to contribute to the economic feasibility of cellulosic biofuel technologies [5, 14–16]. Lignocellulosic feedstocks are composed of cellulose, hemicellulosic polysaccharides and lignin [17]. High contents of cellulose and hemicellulosic polysaccharides are desirable, as these constituents can be hydrolyzed and subsequently fermented to produce biofuels. Lignin, on the other hand, cross-links to hemicellulosic polysaccharides and forms a highly impermeable and complex matrix that shields cell wall polysaccharides from degradation, and impedes the extraction of fermentable sugars from the cell wall [18–21]. Genotypic variation in cell wall composition has been reported in *M. sinensis* and *M. sacchariflorus*, providing ample scope for improving biomass quality in these species through breeding [22, 23].

Compared to annual crops, progress in breeding of perennials, such as miscanthus, is slowed-down by the need to evaluate genotype performance in multi-year field trials. Miscanthus typically matures in 3 years and selection at a premature stage, specifically during its first year of establishment, has proven unreliable [24]. Therefore, the application of marker-assisted selection could substantially increase the efficiency of breeding in miscanthus, as selections could be done at the seedling stage using marker data. Genetic maps form the basis for finding marker-trait associations, but their construction in miscanthus is complicated by the large genome size and the high levels of heterozygosity inherent to its obligate outcrossing nature [11, 13]. Nonetheless, a few genetic maps of miscanthus have been published to date [25–29].

So far three of these genetic maps have been used for the identification of quantitative trait loci (QTL) for different traits of interest, but none of these studies focused on biomass quality for biofuel production. The randomized amplified polymorphic DNA (RAPD) marker-based map by Atienza et al. has been used for identification of QTL associated with agronomic performance and combustion quality [30–34]. The simple-sequence repeat (SSR) marker-based map by Swaminathan et al. was used for the identification of QTL associated with agronomic performance [35]. This map was recently extended with simple nucleotide polymorphism (SNP) markers, obtained through restriction site-associated DNA (RAD) sequencing, and was used for the identification of QTL underlying the zebra stripe phenotype that is desirable for the use of miscanthus as an ornamental grass [29]. Currently, no marker-trait associations have been reported in miscanthus for traits relating to cell wall composition or biomass quality for the production of cellulosic biofuels.

Here we report the construction of a new genetic map for *M. sinensis* using SNP markers obtained through a genotyping-by-sequencing (GBS) approach. The mapping population used in this study segregates for biomass quality traits, as it was derived from a cross between two parental lines with contrasting cell wall composition. The objectives of this study were (1) to detect QTL for biomass composition and quality in miscanthus regarding its use as a lignocellulose feedstock for biofuel production and (2) to align marker sequences to the sorghum reference genome to facilitate cross-species comparisons.

## Methods

### Mapping population

A mapping population of 182 F1 progeny was generated by crossing two *M. sinensis* genotypes with contrasting cell wall composition. The male parent, hereafter referred to as P1, was a genotype (H0227) originating from the miscanthus collection of Wageningen University and Research (WUR). The female parent, hereafter referred to as P2, was derived from a cross between two genotypes from the BIOMIS mapping population (H0012 × H0163) described by Atienza et al., [25]. Both H0012 and H0163 (grandparents) were also included in the field trial and are hereafter referred to as G1-P2 (H0012) and G2-P2 (H0163), respectively. A random sample of seeds was sown in August 2011 in trays in a heated greenhouse; seedlings were subsequently potted and raised to vigorous plants by the end of the winter of 2011/2012. These were split by the end of May 2012 into four roughly equally sized clonal pieces (ramets). Three ramets of each genotype were immediately used to establish a field trial in May 2012; one spare ramet per genotype was potted to replace possible fall-outs. The trial was located at an experimental site of WUR at Wageningen (The Netherlands) and had a randomized block design with the individual ramets used as experimental units. The ramets were planted in rows with a distance between and within rows of 75 cm. The trial was surrounded by two rows of medium-sized *M. sinensis* plants in order to minimize possible border effects. In the second and third growth season, heading date was scored per plant. At the end of the second and third growth season (December 2013 and 2014), all plants were harvested separately, dried to constant weight using ventilated air (dm% ~ 92%) and weighed. A random sample of each plant was subsequently taken, from which leaves and inflorescences were separated from the stem material. The stem fraction of each plant was then chopped into ~2 cm chips, and air-dried at 60 °C for 72 h in a forced-air oven. Stem samples (*n* = 186 genotypes × 3 replicates × 2 years = 1104, minus fall-outs) were ground using a hammer mill with a 1-mm screen and used for biomass quality analyses.

### Biomass quality analysis

Neutral detergent fiber (NDF) and acid detergent fiber (ADF) contents of stem dry matter were determined by detergent fiber analysis using an ANKOM 2000 Fiber Analyzer (ANKOM Technology Corporation, Fairpoint, NY). Acid detergent lignin (ADL) contents were determined after 3-h hydrolysis of the ADF residue in 72% H_2_SO_4_ with continuous shaking. All analyses were performed in triplicate and fiber fractions were expressed in gram per kg dry matter. Fiber fractions were used to calculate the concentrations (in g/kg dm) of cell wall (NDF), cellulose (CEL, equals ADF – ADL), hemicellulosic polysaccharides (HEM, equals NDF – ADF) and acid detergent lignin (ADL) on a dry matter basis. The residual NDF material of the replicated fiber analyses was pooled per sample and used for the determination of neutral sugar and Klason lignin (KL) content as described previously [36]. Briefly, 30 mg of NDF material was hydrolysed for 1 h in 0.3 ml 72% H_2_SO_4_ at 30 °C, after which the acid concentration was diluted to 4% and samples were autoclaved for 60 min at 121 °C. Autoclaved samples were cooled and centrifuged, after which the supernatant was used for determination of glucose (GLU), xylose (XYL) and arabinose (ARA) contents using high performance anion exchange chromatography (HPAEC) on a Dionex system (Dionex, Sunnydale, CA) equipped with a CarboPac PA1 column and a pulsed amperometric detector. The pellet remaining after centrifugation was vacuum-filtered through a pre-weighed glass fibre filter (AP25, Fischer Scientific, Loughborough, UK). The residue was dried overnight at 103 °C and weighed for the determination of KL.

Separate analyses of ground stem samples were performed for the characterization of saccharification efficiency by two different methods. The first method was used for the high-throughput, small-scale quantification of the rate of glucose release during enzymatic hydrolysis of hot-water pretreated samples, as described previously [37]. The release of glucose was expressed as the concentration in nmol of reducing sugars released per mg of biomass per hour of digestion; hereafter referred to as saccharification rate (SacR). The second method was aimed at quantifying the final yield of fermentable sugars using a highly controlled lab-scale alkaline pretreatment and enzymatic saccharification setup, as described by van der Weijde et al. [36]. The released amounts of glucose and xylose are expressed either (1) as a weight percentage of the amount of glucose and xylose present in the untreated sample as determined by neutral sugar analysis (i.e., referred to as glucose conversion (GC) or xylose conversion (XC)) or (2) as a weight percentage of the amount of cellulose and hemicellulose present in the untreated sample as determined by fiber analysis (i.e., referred to as cellulose conversion (CC) and hemicellulose conversion (HC)).

To allow high-throughput analysis of all biomass quality traits we used near-infrared spectroscopy (NIRS) technology. Multivariate prediction models that combined near-infrared (NIR) spectral data and biochemical data were developed for all traits except for SacR. Near-infrared absorbance spectra of stem samples were obtained using a Foss DS2500 near-infrared spectrometer (Foss, Hillerød, Denmark) and processed by weighted multiplicative scatter correction and mathematical derivatization and smoothing treatments (2,6,4,1) using WinISI 4.9 statistical software (Foss, Hillerød, Denmark). Different prediction models were developed for different traits, depending on the number of samples that could be biochemically analyzed and on the availability of existing data for creating robust prediction models (containing a range of miscanthus samples from different experiments) (Table 1). All models contained at least 140 samples from the first growing season of the mapping population. The quality of the prediction models was validated using the squared Pearson coefficient of correlation (*r* ^*2*^) between predicted and biochemical data and by evaluating for these samples the standard error of cross-validation (SECV) for each of the traits (Table 1). Subsequently, the developed prediction models were used to determine biomass composition and conversion efficiency of all 1104 stem samples (minus fall-outs).

**Table 1:** Summary of calibration and cross-validation statistics of mPLS models used for the prediction of biomass quality traits

	Calibration		Cross-validation								
Constituent	# Samples	SECV	# Samples	Chemical analysis			NIRS prediction			*r2*	SEP
				Mean	Min	Max	Mean	Min	Max		
NDF (g/kg dm)	510	8.08	162	880.36	799.98	928.23	880.62	814.19	923.73	0.94	6.15
ADF (g/kg dm)	512	10.54	162	552.73	478.13	632.17	553.35	488.74	629.97	0.85	9.39
ADL (g/kg dm)	491	9.63	162	66.09	40.71	112.18	66.42	43.19	105.86	0.85	8.04
KL (%ndf)	116	0.78	135	13.89	11.05	17.59	13.88	11.76	16.06	0.62	0.95
ARA (%ndf)	249	0.20	249	2.81	1.97	3.57	2.81	2.33	3.38	0.50	0.22
XYL (%ndf)	245	1.22	245	31.10	25.96	36.48	31.16	26.15	35.88	0.78	1.24
GLU (%ndf)	250	2.01	250	51.08	44.36	56.64	51.10	47.02	54.40	0.30	2.04
CC (%)	413	3.07	158	40.14	28.33	52.94	40.38	29.90	46.31	0.73	3.26
HC (%)	408	0.39	158	22.20	17.87	27.03	21.97	19.98	23.40	0.37	1.60
GC (%)	157	3.61	157	47.75	35.50	61.40	47.81	34.60	55.50	0.49	3.80
XC (%)	158	1.83	158	28.61	33.60	22.60	28.59	25.60	31.20	0.28	1.90

SECV = standard error of cross-validation in the set of calibration samples, *r* ^2^ = coefficient of determination between biochemical data and NIRS predicted data; SEP = standard error of prediction

### Genotyping-by-sequencing

Genomic DNA from young leaf tissues was extracted following a CTAB based protocol [38]. DNA concentration and quality were checked using a NanoDrop ND-1000 spectrophotometer (Thermo Fisher Scientific, Waltham, MA) and standardized using a Qubit fluorometer (Thermo Fisher Scientific, Waltham, MA). DNA integrity was confirmed on 1% agarose gels. Libraries were prepared for GBS using the restriction endonuclease ApeKI (five-cutter) to digest the genomic DNA for complexity reduction. Each digested DNA sample was ligated to a set of uniquely barcoded sequencing adaptor pairs, following PCR amplification with adapter-specific primers, and amplicons between 300 and 500 bp were extracted from an agarose gel and sequenced in four single lanes of Illumina HiSeq2000 using a 100 bp paired-end protocol. DNA digestion, adapter ligation, library construction, and sequencing were carried out by the Beijing Genomics Institute (BGI), China.

The de-multiplexed sequence reads obtained from BGI were filtered by removing those reads that did not start with the 5’-CWCG-3’ site pattern, typically resulting from ApeKI digestion, or that contained undefined (‘N’) nucleotides. Reads were right-trimmed to a length of 82 nucleotides and clustered in order to count the number copies per unique read sequence. Note that this clustering was not only done for each sample individually, but also separately for the forward and reverse reads. Only unique reads that occurred at least four times were kept. Unique reads from all samples were jointly clustered using the RADSNP program (RADNPGTv1.1 package, BGI, China). Our initial approach to classify genotypes was to assign a genotypic score to the studied genotypes with a cluster size of at least five reads by applying a set of classification rules to separate clustered reads. The first classification rule was that if the genotype had a frequency of 0.8 or higher for the most abundant read in the cluster, this was considered to be present in homozygous condition. The second classification rule was applied when the two most abundant reads in a cluster if both had frequencies of at least 0.2. The genotype was then classified to be heterozygous. If for a particular cluster neither rule 2 nor 3 held true, no genotypic assignment was given. Unfortunately this approach did not result in acceptable data for map construction, because the average cluster size was too small to allow for a proper genotypic classification due to insufficient sequencing depth. Therefore we refrained from this approach and focused on segregation analyses for single reads. The number of reads for each selected sequence was in this case the basis for genotypic classification using a dominant way of scoring. Genotypes with one or more reads were considered to be either homozygous dominant or heterozygous for this short-sequence marker, whereas the ones showing no reads were supposed to be homozygous recessives. A missing value was assigned to genotype-marker combinations when both the number of reads for this marker over genotypes as well as the average number reads over all markers for a genotype was low. This was done to prevent misclassification of genotypes.

### Map construction

A genetic map was constructed following the two-way pseudo test-cross strategy [39], using the dominantly scored SNP markers. To this end, suitable markers were first filtered out of all available markers (49102) based on segregation ratio, with only uniparental single-dose markers, i.e., markers that segregated in a 1:1 ratio in the population, used for further analysis. A total of 1145 markers remained and were coded according to segregation type following the coding scheme for cross pollinated populations as used in JoinMap [40]. Male simplex × female nulliplex markers were classified as *lm × ll*, while male nulliplex × female simplex markers were classified as *nn × np*. Markers were imported into JoinMap 4.1 (Kyazma, Wageningen, Netherlands) and after elimination of segregation distorted markers and markers that had high similarity (>0.99) to other markers, a total of 1003 markers were used for linkage analyses. These markers were separated into linkage groups using JoinMap grouping analysis with a maximum recombination threshold of 0.25 and a minimum independence logarithm of odds (LOD) score of 2. Markers resolved into 33 linkage groups, 16 linkage groups for the male map and 17 linkage groups for the female map. Marker order within each linkage group was then determined using Haldane’s regression mapping algorithm in JoinMap with a maximum recombination threshold of 0.40 and a minimum independence logarithm of odds (LOD) score of 1. This procedure built a map by adding loci one by one, starting from the most informative pair of loci. Each locus was added at its best position according to a goodness-of-fit measure or removed again until all loci are handled two times. The male map spanned 2139.7 cM and consisted of 242 markers with a median inter-marker spacing of 8.0 cM. The female map spanned 2479.5 cM and consisted of 322 markers with a median inter-marker spacing of 6.7 cM.

### Statistical analysis and QTL mapping

General analyses of variance (ANOVA) were performed to determine the significance of genotype differences (*p* < 0.05) in the mapping population for cell wall composition and saccharification efficiency. Variance analyses were performed separately for both growing seasons, taking into account the randomized complete block design of the trial. Estimates of genotypic (σ_g_^2^) and residual (σ_e_^2^) variance were used to calculate broad sense heritability (*h* ^*2*^) estimates following *h* ^*2*^ = σ_g_^2^/(σ_g_^2^ + σ_e_^2^). To visualize associations amongst traits, a principal component analysis was performed on genotype means for all traits evaluated in both growth seasons. Origin centered, normalized scores for the first two principal components were plotted in a principal-component biplot. All statistical analyses were performed using Genstat for Windows, 18th edition software package (VSN International, Hemel Hempstead, UK).

Quantitative trait loci (QTL) analysis was performed with MapQTL 6.0 (Kyazma, Wageningen, Netherlands) using a maximum likelihood mixture model. An interval mapping approach was used with a step size of 1.0 cM. Significance of a QTL was called based on a LOD score higher than a genome-wide significance threshold based on 1000 permutations [41], which was determined to be 3.561 for the male and 3.655 for the female map. One-LOD and two-LOD support intervals were determined to show the uncertainty on the QTL position. The percentage of variance explained (PVE) by the QTL was calculated by 100 × ([residual variance with no QTL fitted – residual variance with QTL fitted]/population variance) [42].

## Results and discussion

### Genotypic variation for biomass quality traits

Significant heritable variation was observed in the mapping population for all stem biomass quality traits determined after the second and third growth seasons, as shown by the population statistics and parental and grand-parental values summarized in Table 2. Cell wall material (NDF) comprised the largest fraction of biomass and ranged from ~815 to 911 g/kg dm in the second and from ~877 to 918 g/kg dm in the third growth season. The main cell wall components were CEL and HEM, with variation in the population in the second growth season ranging from ~446–527 and ~304–365 g/kg dm, respectively. In the third growth season plants had on average higher CEL and lower HEM contents compared to the second growth season and ranged from ~474–532 and ~282–345 g/kg dm, respectively. Particularly large variation in cell wall glucose content (GLU) was also found, ranging from ~35 to 50% of the cell wall fraction in the second and from ~21 to 39% in the third growth season.

**Table 2:** Descriptive statistics of the mapping population for biomass growth and quality characteristics relevant to the use of miscanthus for biofuel production

	Growing	P1	G-P1	G-P2	Population statistics			
Trait	season	(*H0227*)	(*H0012*)	(*H0163*)	Mean	Range	LSD	*h* ^*2*^
NDF (g/kg dm)	1	*	899.8	838.7	880.0	81.53–91.13	16.5	0.63
	2	911.5	889.0	899.1	903.0	87.69–91.76	10.4	0.39
ADF (g/kg dm)	1	*	576.8	500.2	546.0	49.09–60.68	18.8	0.63
	2	584.7	585.8	593.1	594.0	54.94–62.87	19.7	0.40
CEL (g/kg dm)	1	*	506.2	453.8	482.0	44.65–52.72	15.3	0.62
	2	499.3	495.4	497.3	502.0	47.41–53.21	16.6	0.40
HEM (g/kg dm)	1	*	323.0	338.5	334.0	30.45–36.47	11.0	0.72
	2	326.8	303.1	306.0	309.0	28.21–34.77	14.2	0.55
ADL (g/kg dm)	1	*	70.7	46.4	62.0	4.2–8.18	6.5	0.73
	2	85.4	90.4	95.8	90.0	7.48–11.03	6.3	0.59
CEL/cw (% NDF)	1	*	56.2	54.1	55.1	51.4–57.9	1.1	0.72
	2	54.8	55.7	55.3	55.5	52.9–58	1.4	0.47
HEM/cw (% NDF)	1	*	35.9	40.4	38.0	33.4–42.5	1.4	0.68
	2	35.8	34.1	34.0	34.3	31.4–38.8	1.7	0.49
ADL/cw (% NDF)	1	*	7.8	5.5	7.0	4.9–9.2	0.7	0.72
	2	9.4	10.2	10.7	10.0	8.3–12.3	0.7	0.62
KL (% NDF)	1	*	13.5	14.0	13.7	12.1–15.2	0.5	0.72
	2	12.3	13.9	14.1	14.1	11.5–15.7	0.8	0.65
GLU (% NDF)	1	*	52.3	51.7	51.7	50.0–53.2	0.7	0.64
	2	52.4	51.2	49.3	50.1	48.0–52.2	0.9	0.50
XYL (% NDF)	1	*	33.7	33.2	33.2	30.5–36.0	0.9	0.65
	2	28.8	28.3	30.4	29.2	27.1–32.0	1.1	0.52
ARA (% NDF)	1	*	2.8	2.7	2.7	2.5–3.2	0.1	0.69
	2	3.0	2.8	3.2	2.9	2.6–3.4	0.2	0.60
CC (% CEL)	1	*	40.1	46.2	41.2	36.9–46.2	1.7	0.66
	2	34.7	36.0	33.2	25.3	30.2–39.1	3.1	0.35
HC (% HEM)	1	*	22.8	21.6	22.0	21.3–22.6	0.4	0.46
	2	20.6	21.7	21.1	13.7	20–22.1	1.5	0.31
GC (% GLU)	1	*	53.2	48.8	48.8	42.3–54.8	1.9	0.74
	2	45.2	41.6	38.4	40.1	32.5–45.7	3.2	0.47
XC (% XYL)	1	*	30.2	28.6	28.5	26.83–31.35	0.7	0.63
	2	29.7	28.4	27.5	27.9	26.7–30.5	0.9	0.54
SacR ()	1	*	14.4	21.3	18.1	10.7–24.3	3.7	0.53
HD (Julian days)	1	*	228.1	261.7	228.2	213.7–257.4	9.5	0.63
	2	228.3	213.3	224.3	208.8	196.3–225.7	6.4	0.72

LSD = least significant difference (*p* = 0.05), *h* ^2^ = heritability

* missing value

Variation in ADL ranged from ~42 to 82 g/kg dm in the second and from ~75 to 110 g/kg dm in the third growth season. ADL/cw and KL ranged from ~5–9% and ~12–15% of the cell wall in the second and from ~8–12% and 12–16% in the third growth season, respectively. Variation in lignin content is of particular interest for improving biomass quality of miscanthus, and variation in both ADL and KL was extensive. KL values are higher than ADL values, as during the quantification of ADL, detergents are used that likely dissolve a fraction of the total lignin. However, KL values might overestimate lignin as it is more likely to be contaminated with protein [43, 44]. Both methods provide different but valuable insights into biomass quality [36].

The mapping population also harbored extensive variation in conversion efficiency. Particularly for SacR, GC and XC, considerable variation was observed among genotypes. Variation in SacR ranged from ~11 to 24 nmol reducing sugars per mg biomass per hour. Variation in GC ranged from ~42 to 55% in the second and from ~33 to 46% in the third growth season. These ranges are comparable with the ranges observed in other highly diverse sets of miscanthus genotypes [23, 36, 45–47], indicating that variation in conversion efficiency in this population created by crossing two highly compositionally distinct parents is substantial. Conversion efficiency values in the third growth season were substantially lower than those found in the second, which is presumably associated with the increase in lignin content observed with increasing plant age (Table 2).

Genotype performance for most of the evaluated traits was highly reproducible across replicated blocks. As a result, for most traits a high heritability (*h* ^*2*^ > 0.5) was observed, with the highest heritability for quality traits across years observed for lignin (ADL/cw) (*h* ^*2*^ = 0.62–0.72). For all traits, heritability estimates in the third growth season were reduced compared to those in the second. The lower heritabilities in the third growth season can be caused by the lower range of observed genetic variation, environmental effects, errors in biochemical analyses, and/or lower NIRS prediction accuracy. The heritability estimates for compositional and conversion efficiency characters are consistent with values observed by others in maize and sorghum mapping studies [48–50].

**Fig. 1:**
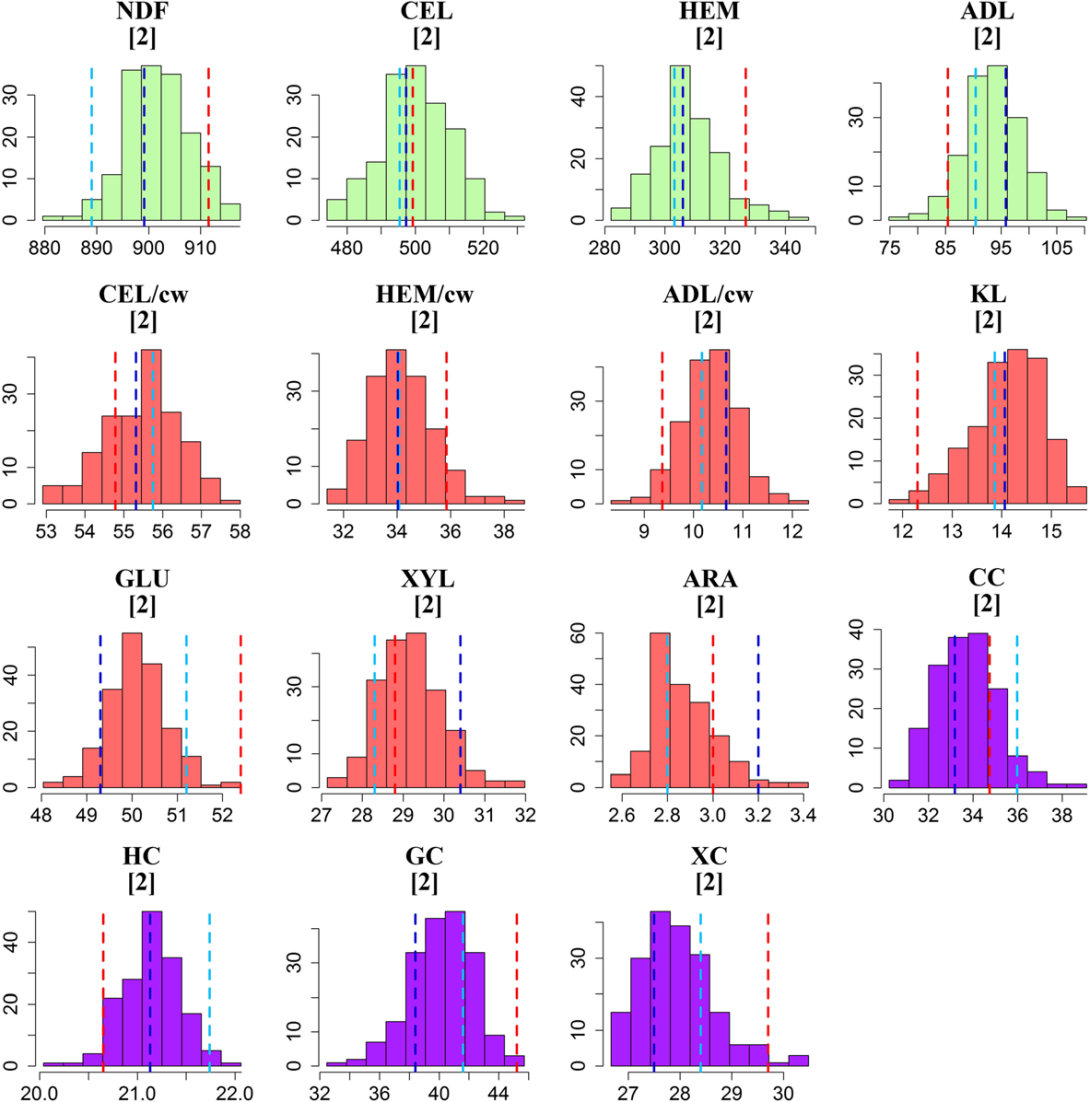
Histograms displaying the frequency distributions of genotype values for stem composition and conversion efficiency characters after the third growth season. Unit of the y-axis is the number of genotypes, while the unit of the x-axis depends on the unit of the plotted trait. Lines represent (grand) parental values, with *red line* depicting P1, the *light-blue line* depicting G1-P2 and the *dark-blue line* depicting G2-P2

Frequency distributions of all traits evaluated in the third growth season were reasonably uniform and showed continuous unimodal histograms (Fig. 1). For all traits, with the exception of CEL, parental and grand-parental performance were contrasting and for most traits population variation extended beyond parental and grand-parental values in both directions. For KL and GLU, the performance of P1 was very near the low-end population extreme; hence genetic variation leading to concentrations lower than those observed for P1 for these traits is not expected in this population.

**Fig. 2:**
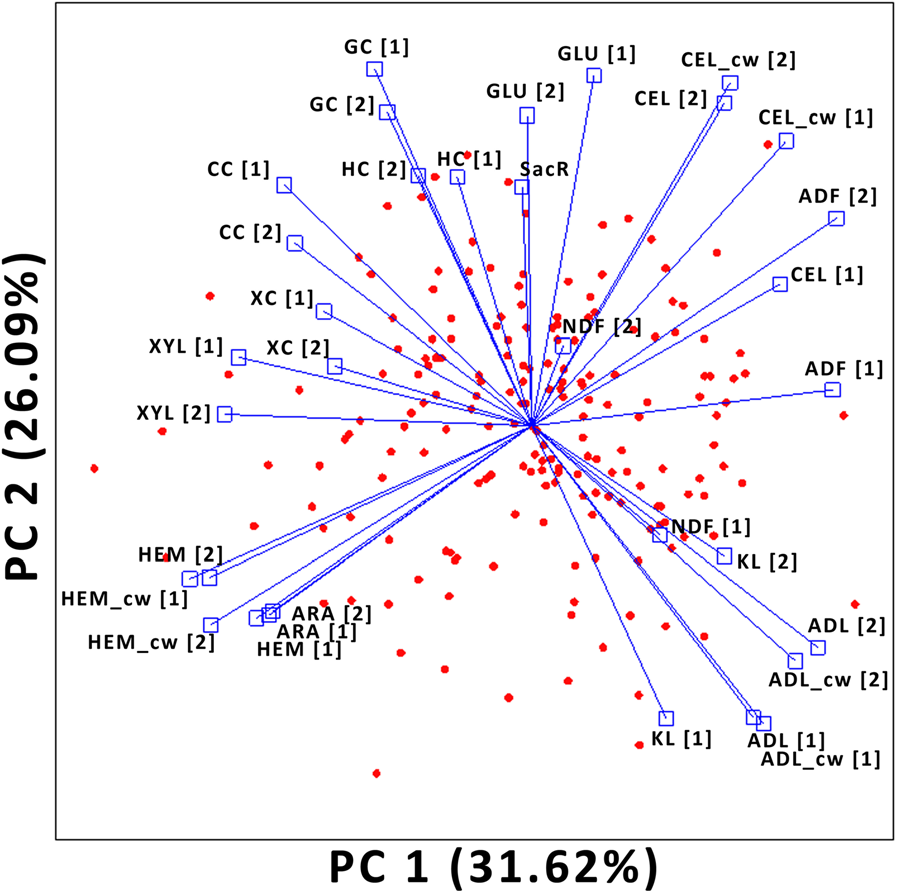
Principal component biplot displaying variation in cell wall composition and conversion efficiency harbored within the mapping population. *Red dots* are genotype mean scores. Trait names designated with ‘[1]’ were scored after the second growth season of the population, while those designated with ‘[2]’ were scored after the third growth season. Vectors represent traits, with the angle between a vectors and the principal component axis proportional to the contribution of the corresponding trait to those principal components. The length of vectors is proportional to the variance observed for the corresponding trait and the angle between vectors is proportional to the correlation among characters

Principal components analysis revealed that approximately 58% of the observed genotypic variation in biomass quality resolved into two composite variables (Fig. 2). The first principal component summarized 32% of the observed genotypic variation and predominantly discriminated genotypes based on differences in the content of cellulosic and hemicellulosic polysaccharides. The second component, which summarized 26% of observed variation, discriminated genotypes mostly based on differences in lignin and conversion efficiency characters. As the angle between vectors is representative of correlations between traits, from this plot it can be deduced that the different conversion efficiency characters are positively associated to each other and negatively associated with lignin. It is also evident that SacR was more strongly correlated with the content of cellulosic polysaccharides than was cellulose conversion. These trait associations are consistent with other reports on miscanthus biomass composition and quality for biofuel production [36, 45, 47].

### Synteny with *Sorghum bicolor* and coding of linkage groups

The DNA sequences of the mapped markers were aligned to the *Sorghum bicolor* (L.) Moench genome (version ‘sbi1’) from the Plant Genome Database using NCBI BLASTN [51]. Only hits with an identity score greater than 85% and an alignment length of at least 50 nucleotides were retained and used to label the miscanthus linkage groups according to which sorghum chromosome the markers in each linkage group mapped (Additional file 1: Figure S1). Linkage groups of the female map were designated by the corresponding *Sorghum bicolor* chromosome numbers, followed by an ‘a’ or ‘b’, as well as linkage groups of the male map, but followed by a ‘c’ or ‘d’. These suffixes were randomly appointed to the two homologous miscanthus linkage groups of each map that are syntenic to each sorghum chromosome, as the genome of *M. sinensis* consists of two sub-genomes with a high level of synteny to the sorghum genome [26, 27]. In both, the male and the female map, there was one linkage group that aligned with two sorghum chromosomes; these groups were designated ‘4b7b’ and ‘4d7d’. The occurrence of this phenomenon in miscanthus has been reported previously and is ascribed to an ancient chromosome fusion or translocation event between two miscanthus chromosomes syntenic to sorghum chromosomes 4 and 7. This event explains why miscanthus has a basic chromosome number of 19 and not 20 (twice the basic chromosome number of sorghum) [27, 28].

### QTL mapping of miscanthus biomass quality traits

**Fig. 3:**
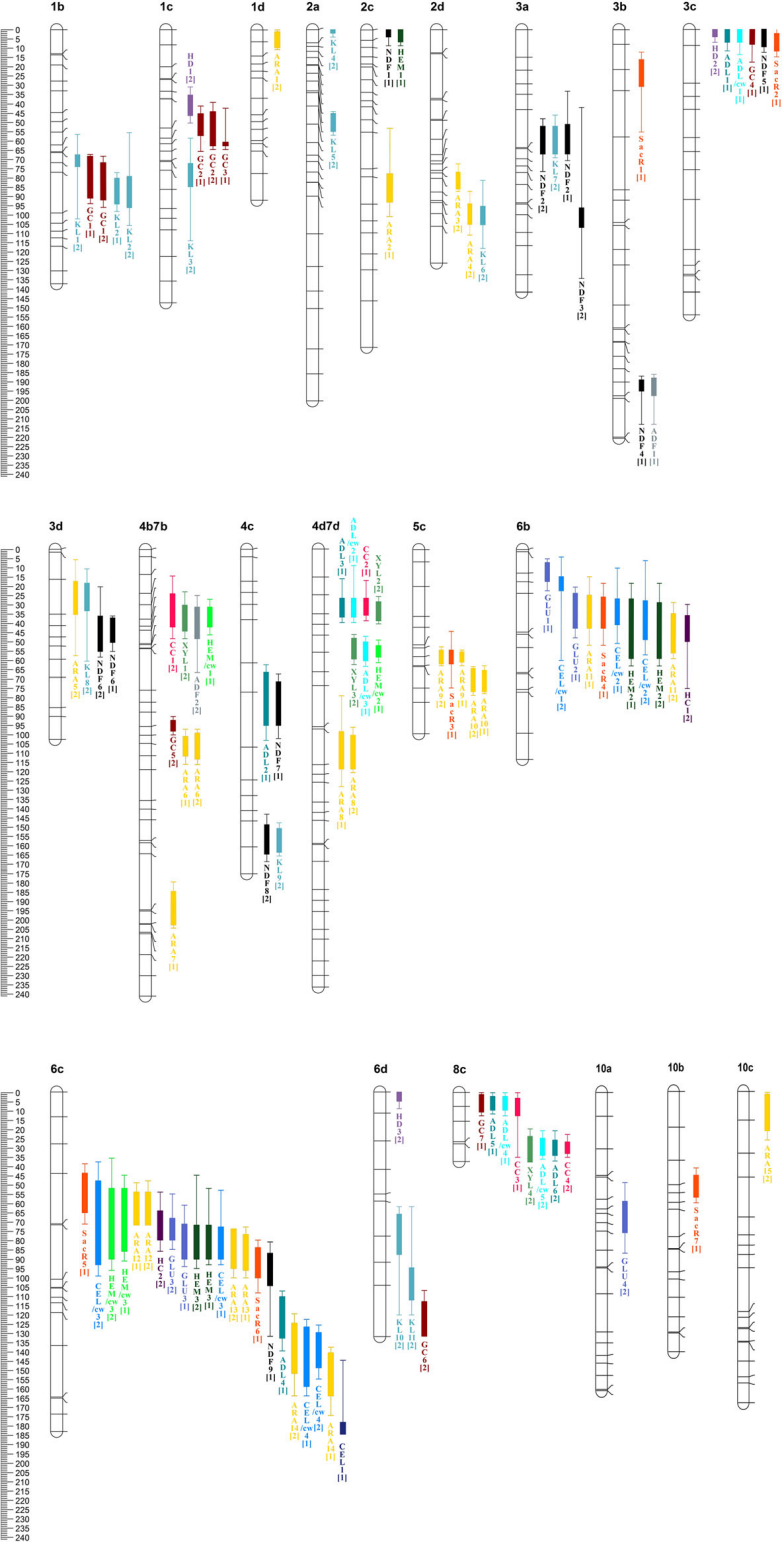
Distribution of QTLs identified for biomass composition and conversion efficiency across 19 linkage groups of two genetic maps of *M. sinensis*. Linkage groups designated with ‘a’ or ‘b’ originate from the female map, while those designated with ‘c’ or ‘d’ originate from the male map. QTLs designated with ‘[1]’ were observed in the second growth season of the population, while those designated with ‘[2]’ were observed in the third growth season

QTL analysis was performed to investigate associations between genomic regions and stem composition and conversion traits. In a combined QTL analysis carried out on the male and female map simultaneously a total of 86 QTLs were found to be associated with cell wall composition and conversion efficiency characters with LOD scores ranging from 3.58 to 9.02 (Table 3). Heterozygosity was uncovered in 58 loci of the male parent and 28 loci of the female parent, but these may be partly the same loci if the male and the female map would be combined. Twenty out of 86 QTLs were found in both growth seasons. In the combined analysis, significant QTLs were located across 21 out of the total of 33 male and female linkage groups (Fig. 3). For several traits, QTLs were observed to be present in roughly the same genomic position in presumably homeologous linkage groups in both parental maps (e.g., QTLs for ARA on groups 2c and 2d).

**Table 3:** Observed QTLs for stem cell wall composition and conversion efficiency characters

QTL	Year	LG	Position	1-LOD support interval	2-LOD support interval	LOD	PVE
			(cM)	(cM)	(cM)		
NDF 1	2013	2c	0.0	Start - 4.0	Start - 8.7	5.77	13.9
NDF 2	2014	3a	61.0	52.0–67.1	48.0–76.5	4.98	13.6
NDF 2	2013	3a	64.1	51.0–67.1	33.3–70.6	3.96	9.5
NDF 3	2014	3a	101.8	96.1–106.8	41.9–134.3	3.70	10.3
NDF 4	2013	3b	190.1	188.9–195.1	186.9–213.0	4.33	10.4
NDF 5	2013	3c	4.0	Start - 9.6	Start - 12.6	5.73	15.0
NDF 6	2014	3d	40.1	36.1–55.3	20.3–58.3	4.00	10.3
NDF 6	2013	3d	44.2	37.1–50.4	36.1–55.3	4.72	12.9
NDF 7	2013	4c	81.1	71.4–95.1	67.4–102.1	3.77	11.0
NDF 8	2014	4c	156.7	148.7–164.7	143.0–168.7	3.58	10.2
NDF 9	2013	6c	98.8	86.8–104.0	80.8–131.0	3.59	10.1
ADF 1	2013	3b	191.1	187.9–197.5	185.9–213.0	3.81	9.6
ADF 2	2014	4b7b	40.7	31.9–48.7	25.8–66.6	3.74	9.3
CEL 1	2013	6c	181.9	176.9–183.3	143.8–183.3	3.98	10.8
CEL/cw 1	2014	6b	18.5	14.8–22.5	4.2–60.1	4.44	11.2
CEL/cw 2	2013	6b	33.8	26.8–40.8	10.2–50.9	7.54	23.0
CEL/cw 2	2014	6b	36.8	27.8–48.9	6.2–57.1	4.78	14.6
CEL/cw 3	2014	6c	81.8	48.1–92.8	38.0–98.8	4.85	15.8
CEL/cw 3	2013	6c	82.8	72.8–89.8	53.1–92.8	9.02	29.1
CEL/cw 4	2014	6c	136.0	129.0–147.8	125.0–153.8	4.14	11.2
CEL/cw 4	2013	6c	147.8	126.0–157.8	122.0–162.8	4.61	16.5
HEM 1	2013	2c	2.0	Start - 6.7	Start - 8.7	3.78	10.3
HEM 2	2013	6b	34.8	26.8–59.1	18.5–63.1	4.88	15.5
HEM 2	2014	6b	48.9	28.8–59.1	18.5–63.1	4.16	10.7
HEM 3	2014	6c	79.8	71.8–89.8	45.1–94.8	6.06	18.5
HEM 3	2013	6c	81.8	71.8–89.8	52.1–92.8	7.81	25.6
HEM/cw 1	2013	4b7b	39.7	31.9–42.5	27.8–46.7	4.40	11.6
HEM/cw 2	2013	4d7d	55.8	52.5–58.8	49.5–61.8	3.64	9.3
HEM/cw 3	2014	6c	79.8	52.1–89.8	36.0–95.8	5.11	16.1
HEM/cw 3	2013	6c	74.8	52.1–85.8	45.1–90.8	4.61	12.7
ADL 1	2013	3c	2.0	Start - 7.0	Start - 11.6	5.18	13.4
ADL 2	2013	4c	74.4	66.4–95.1	62.4–103.1	3.87	10.8
ADL 3	2013	4d7d	31.7	26.7–37.0	16.1–4Start	3.76	11.4
ADL 4	2013	6c	116.7	109.8–132.0	106.8–138.8	3.81	10.2
ADL 5	2013	8c	7.0	2.0–9.5	Start - 11.5	5.57	14.4
ADL 6	2014	8c	28.8	25.6–33.8	20.6–36.8	5.25	13.4
ADL/cw 1	2013	3c	2.0	Start - 7.0	Start - 13.6	4.45	11.6
ADL/cw 2	2013	4d7d	31.7	26.7–37.0	9.0–4Start	3.79	11.4
ADL/cw 3	2013	4d7d	55.5	50.5–60.8	47.5–63.8	3.78	9.9
ADL/cw 4	2013	8c	6.0	2.0–9.5	Start - 12.5	5.51	14.8
ADL/cw 5	2014	8c	28.8	24.6–33.8	20.6–35.8	5.61	14.2
KL 1	2014	1b	71.7	68.2–74.7	57.2–102.9	5.23	12.8
KL 2	2013	1b	87.9	80.9–94.9	77.9–98.9	6.45	21.0
KL 2	2014	1b	87.9	79.9–96.9	56.2–106.5	4.84	16.3
KL 3	2014	1c	77.8	72.1–84.8	58.5–114.0	3.69	9.9
KL 4	2014	2a	0.0	Start - 2.0	Start - 4.0	4.66	11.4
KL 5	2014	2a	49.1	45.1–54.9	44.1–56.9	5.98	16.7
KL 6	2014	2d	102.3	95.3–105.3	81.3–118.1	5.58	15.0
KL 7	2014	3a	63.0	52.0–67.1	46.0–69.1	4.07	10.0
KL 8	2014	3d	25.3	18.3–33.3	10.6–60.5	4.30	14.6
KL 9	2014	4c	157.7	150.7–163.7	147.7–165.7	5.57	15.0
KL 10	2014	6d	76.0	66.0–87.9	62.0–120.3	3.87	10.3
KL 11	2014	6d	100.8	94.8–112.3	62.0–120.3	3.92	12.8
GLU 1	2013	6b	12.2	7.2–17.5	5.2–22.5	8.22	19.4
GLU 2	2013	6b	33.8	23.8–42.8	20.5–47.9	6.75	19.8
GLU 3	2013	6c	80.8	71.2–89.8	61.1–93.8	6.84	22.5
GLU 3	2014	6c	71.8	68.1–79.8	55.1–84.8	4.09	9.9
GLU 4	2014	10a	68.6	58.9–75.8	48.7–86.9	4.67	11.6
XYL 1	2014	4b7b	39.7	30.9–44.7	23.8–48.7	4.45	11.6
XYL 2	2014	4d7d	35.0	28.7–39.0	25.7–40.7	4.34	10.5
XYL 3	2014	4d7d	55.8	48.5–59.8	46.5–62.8	4.57	11.3
XYL 4	2014	8c	30.8	23.6–37.4	19.6–37.4	4.29	12.0
ARA 1	2014	1d	6.0	1.0–9.4	Start - 10.4	4.26	11.7
ARA 2	2013	2c	85.2	77.9–93.2	53.4–101.0	3.87	12.1
ARA 3	2014	2d	80.3	77.3–86.3	72.9–87.6	4.24	10.2
ARA 4	2014	2d	100.3	94.3–105.3	87.6–111.1	4.95	12.4
ARA 5	2014	3d	26.3	17.3–35.1	5.6–57.3	3.69	12.9
ARA 6	2013	4b7b	104.7	100.3–110.7	96.5–115.2	4.39	10.9
ARA 6	2014	4b7b	103.3	98.5–112.3	96.5–115.3	4.77	12.8
ARA 7	2013	4b7b	193.3	182.3–200.1	177.3–201.8	4.18	10.8
ARA 8	2013	4d7d	109.1	99.1–119.4	79.6–129.0	4.18	13.1
ARA 8	2014	4d7d	110.1	101.1–119.4	96.8–121.4	3.85	12.1
ARA 9	2013	5c	57.8	55.2–60.8	54.2–62.8	5.17	12.3
ARA 9	2014	5c	57.8	54.2–61.8	52.5–62.8	4.42	10.6
ARA 10	2013	5c	72.2	65.2–76.7	62.8–77.7	4.33	11.6
ARA 10	2014	5c	72.2	64.2–76.7	63.2–78.7	3.79	10.2
ARA 11	2013	6b	33.8	24.8–42.8	14.8–51.9	6.94	21.0
ARA 11	2014	6b	50.9	34.8–56.1	28.8–59.1	6.11	15.2
ARA 12	2013	6c	65.1	54.1–71.8	49.1–71.8	7.57	21.0
ARA 12	2014	6c	65.1	54.1–71.8	48.1–71.8	7.40	21.0
ARA 13	2014	6c	83.8	73.8–94.8	73.8–99.8	7.81	23.8
ARA 13	2013	6c	85.8	76.8–95.8	72.8–99.8	8.55	27.4
ARA 14	2013	6c	149.8	139.8–162.8	136.8–173.3	4.12	15.1
ARA 14	2014	6c	136.0	124.0–150.8	119.0–162.8	4.33	11.0
ARA 15	2014	10c	10.0	1.0–20.7	Start - 25.7	4.47	13.0
SacR 1	2013	3b	23.6	16.0–30.6	12.0–55.0	4.01	11.0
SacR 2	2013	3c	7.0	2.0–11.6	Start - 14.6	6.43	16.1
SacR3	2013	5c	58.8	54.2–61.8	44.0–74.7	3.92	10.2
SacR 4	2013	6b	33.8	25.8–42.8	18.5–51.9	4.31	14.7
SacR 5	2013	6c	55.1	44.0–65.1	39.0–71.2	4.44	15.3
SacR 6	2013	6c	90.8	83.8–99.8	79.8–107.8	5.96	21.9
SacR 7	2013	10b	52.2	44.8–56.7	40.8–59.7	4.19	10.7
CC 1	2014	4b7b	32.9	24.8–42.5	15.5–48.7	4.26	11.4
CC 2	2013	4d7d	30.7	26.7–36.0	17.1–39.0	4.12	12.8
CC 3	2013	8c	7.5	3.0–12.5	Start - 34.8	3.75	9.6
CC 4	2014	8c	28.8	26.6–32.8	22.6–34.8	5.65	14.7
HC 1	2014	6b	44.7	35.8–49.9	29.8–75.2	3.79	9.2
HC 2	2014	6c	71.8	64.1–79.8	54.1–85.8	4.78	11.4
GC 1	2013	1b	76.7	69.2–91.9	68.2–94.9	5.38	13.7
GC 1	2014	1b	83.9	72.7–92.9	69.2–96.9	4.62	14.7
GC 2	2013	1c	55.0	45.3–57.0	41.2–65.5	4.65	13.2
GC 2	2014	1c	54.9	44.2–62.5	39.3–64.5	4.19	11.7
GC 3	2013	1c	61.5	60.5–62.5	42.4–64.5	4.96	12.4
GC 4	2013	3c	0.0	Start - 8.0	Start - 17.6	7.00	9.8
GC 5	2014	4b7b	95.5	91.8–97.5	89.8–99.5	4.62	14.7
GC 6	2014	6d	123.3	113.3 - End	107.3 - End	3.94	14.5
GC 7	2013	8c	7.0	1.0–10.5	Start - 12.5	5.24	13.5
HD1	2014	1c	37.3	35.3–46.3	31.0–50.3	3.93	9.8
HD2	2014	3c	0.0	Start - 4.0	Start - 7.0	3.89	9.4
HD3	2014	6d	0.0	Start - 5.0	Start - 9.0	3.60	8.9

*LG* linkage group, *PVE* percentage variance explained

Out of the 86 QTLs that were observed, 9 were associated with stem cell wall, 5 with cellulose, 6 with hemicellulosic polysaccharides, 22 with lignin and 23 with neutral sugar contents (Table 3).. The QTLs on the male map for ARA were numerous; this high number was likely due to factors such as the inherent imprecision of the QTL mapping approach, marker multicollinearity and the height of the LOD score thresholds used. The large number of QTLs found to be associated with lignin content could be partly explained by the fact that three different lignin characters (ADL, ADL/cw and KL) were evaluated. Notably, QTLs associated with KL did not co-localize with QTLs for ADL or ADL/cw (Fig. 3). Two major-effect QTLs were identified for CEL/cw (CEL/cw 3 and CEL/cw 4) in linkage groups of the male parent, each respectively accounting for 29% and ~17% of the observed genotypic variation during the third growth season (Table 3). These may be interesting targets for further study.

A total of 20 QTLs were found for conversion efficiency characters with LOD-scores ranging from 3.75 to 7.00, among which 7 for SacR, 4 for cellulose conversion, 2 for hemicellulose conversion and 7 for glucose conversion (Table 3).. QTLs for SacR and GC co-localized on linkage group 3c, QTLs for SacR and HC co-localized on linkage groups 6b and 6c (potentially homologous groups) and QTLs for CC and GC co-localized in linkage group 8c. However, many QTLs for the different conversion characters did not co-localize and seem to be independently controlled characters (Fig. 3). On linkage groups 1b, 1c, 3c, 4d7d, 6c, 6d and 8c QTLs for conversion efficiency characters co-localized with QTLs for lignin characters. Particularly strong evidence for co-localization of QTLs for these traits was found on linkage group 1b and 8c, where QTLs for lignin (KL, ADL and ADL/cw) and conversion characters (CC and GC) co-localized in both growth seasons. On linkage groups 4b7b, 4d7d, 6b, 6c and 8c QTLs for conversion efficiency characters co-localized with QTLs for accumulation of hemicellulosic polysaccharides. A big clustering of co-localized QTLs were observed on linkage groups 6b and 6c, possibly indicating the presence of a master-regulator affecting cell wall biosynthesis. QTLs for the same traits co-localized in both clusters, suggesting that 6b and 6c are homologous linkage groups. Several QTLs for conversion efficiency characters did not co-localize with any of the QTLs for compositional characters evaluated in this study (e.g., SacR on 3b and 10b), suggesting that other, unidentified compositional characters are affecting conversion efficiency. One such character, for example, could be the content of hydroxycinnamic acids, such as *para*-coumaric or ferulic acids, which were recently identified as key factors affecting the conversion efficiency of miscanthus biomass [47].

### Comparative analysis of QTL in miscanthus and sorghum

In addition to identifying QTLs for miscanthus biomass composition and conversion characters, an objective of this study was to demonstrate that by aligning the genetic map of miscanthus to the physical map of *Sorghum bicolor*, the exchange of information from genetic studies across species is facilitated and a wealth of information becomes available for the genetic improvement of miscanthus. For this particular objective, the heading date of the genotypes used in this study was scored in both growth seasons, as this is a trait that normally has a high heritability in miscanthus and was previously mapped in miscanthus [35]. Due to the high level of synteny between miscanthus and sorghum, QTL found in one species might have corresponding QTL in homologous regions in the other. In this study, 3 QTLs were identified for heading date, located on linkage groups 1c, 3c and 6d (Table 3). A QTL for heading date on the linkage group that aligns with Sb03 was also identified by Gifford et al.*,* [35] on the same position at the end of the chromosome arm (position 6–9 cM) as HD2 in this study. In addition, a QTL for heading date was consistently reported in sorghum on the end of the chromosome arm of Sb06 [50, 52, 53], which is in accordance with HD3 found in this study.

Similarly, QTLs for NDF are reported in sorghum on chromosomes Sb02, Sb03, Sb04 and Sb06 [50, 54], which may correspond to QTLs for NDF found in this study on the corresponding linkage groups 2c, 3a, 3c, 4c and 6c (Table 3, Fig. 3). The QTL on chromosome Sb03 was reported to have a strong effect and explained a large fraction of the observed variation in a sorghum mapping population [50]. The strong effect of this QTL in sorghum may explain why the presumably corresponding QTL was detected on both the female and the male map in both growth seasons (NDF2 on linkage group 3a and NDF6 on linkage group 3d). QTLs for ADL were identified on Sb03, Sb04, Sb06, Sb07 and Sb08 in sorghum [50, 54], which may correspond to QTLs for ADL in this study, which were observed on all of the corresponding linkage groups (Table 3, Fig. 3). Similar to the clusters of QTLs for different traits that co-localized on miscanthus linkage groups 6b and 6c, a cluster of co-localizing QTLs, including QTLs for cellulose and hemicellulosic polysaccharide accumulation, was observed in sorghum chromosome Sb06 [54]. In a number of genetic studies in sorghum that mapped conversion efficiency characters, QTLs for conversion efficiency repeatedly mapped to chromosome Sb03, Sb04 and Sb07 [55–57]. In this study, QTLs for SacR and GC were located on corresponding linkage groups 3b, 3c, 4b7b and 4d7d. However, several QTLs also mapped to linkage groups that correspond to sorghum chromosome Sb06, for which no QTL associated with conversion efficiency were detected in sorghum so far. These could represent previously unidentified loci affecting conversion efficiency in sorghum.

The fact that (1) several QTLs were identified in both growth seasons and (2) that several QTLs mapped to syntenous chromosomal segments in sorghum provides some indications that these QTLs contain genetic determinants for the traits of interest. Characterization of these QTLs, however, needs further validation. The alignment of this miscanthus genetic map to the *Sorghum bicolor* physical map facilitates the exchange of information between the two species, as well as to other grass species with a syntenic relationship to sorghum. Novel tools, such as the Orphan Crop Genome Browser provide excellent opportunities to exploit such phylogenetic relationships to annotate the genome of miscanthus [58]. Using this tool the regions in the sorghum genome that are homeologous to the QTLs mapped in miscanthus in this study can be easily examined for putative orthologous genes that are reported to affect cell wall compositional characters in crops such as sorghum, maize or rice.

## Conclusions

To our knowledge this is the first report of QTLs for biomass composition and conversion efficiency characters in miscanthus. The large number (86) of identified QTLs highlights the genetic complexity and highly quantitative genetic control of such traits. The alignment of this miscanthus genetic map to the *Sorghum bicolor* physical map facilitates cross-species comparisons of mapped traits and may expedite our understanding of the genetic control of important biomass quality traits in the large, complex and largely unexplored genome of the bioenergy crop miscanthus. These results are a first step towards the development of marker-assisted selection programs in miscanthus to improve biomass quality for biofuel production.

## Additional file

Additional file 1: Figure S1. Synteny map depicting the alignment and localization of *M. sinensis* mapped markers to the Sorghum bicolor (L.) Moench genome. Linkage groups of the female map are designated as ‘a’ or ‘b; linkage groups of the male map are designated as ‘c’ or ‘d’. The position of mapped QTLs is also shown. For each QTL, colored boxes indicate 1-LOD support intervals while extension bars delimit 2-LOD support intervals. (PDF 5499 kb)
