# Supplementary figures and images for: Genetic complexity of miscanthus cell wall composition and biomass quality for biofuels
Source: BMC Genomics. 2017 May 25;18:406. doi: 10.1186/s12864-017-3802-7 (PMC5445440; doi:10.1186/s12864-017-3802-7)

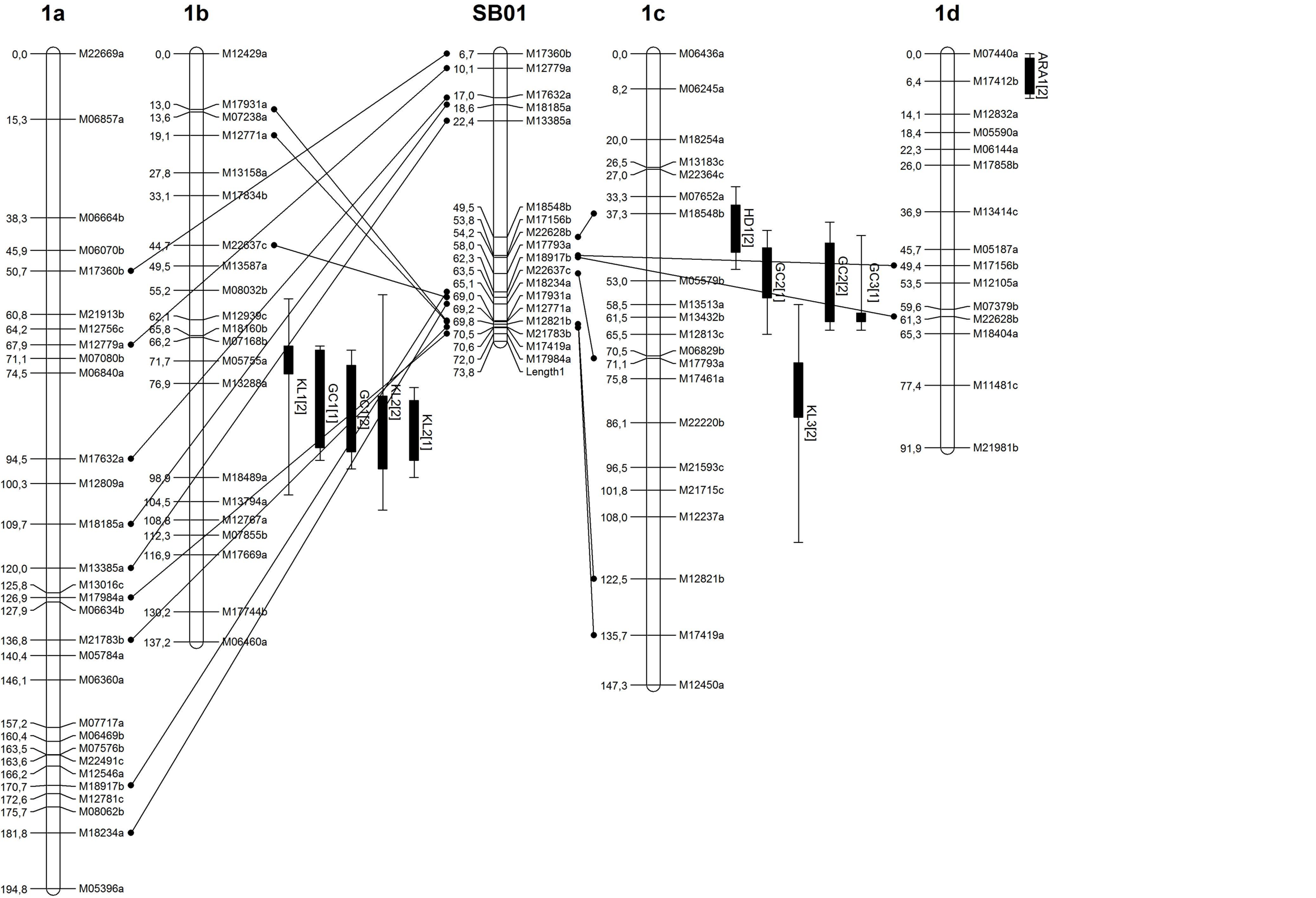

2aR

2b

SB02

2cR

2d

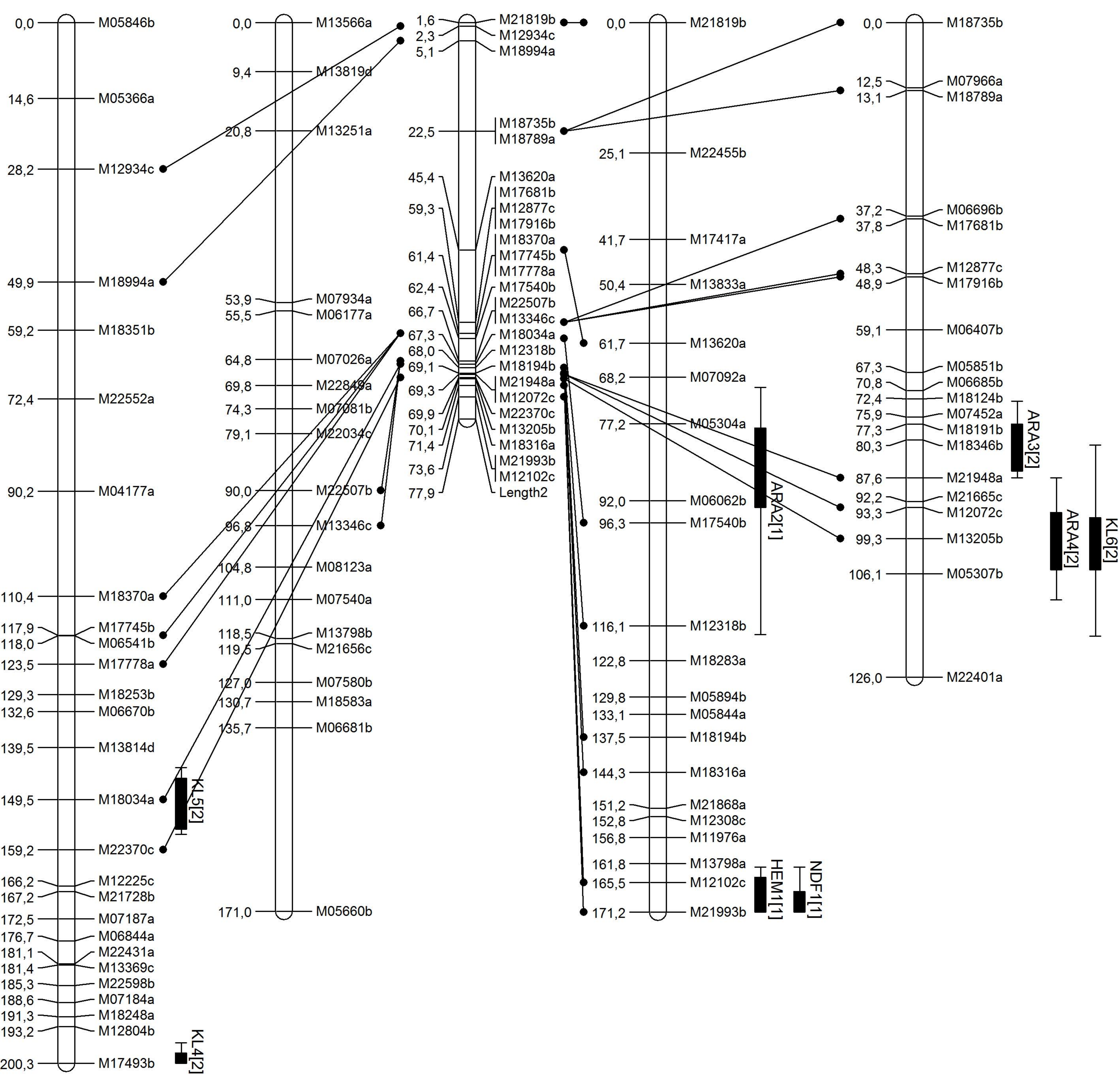

3aR

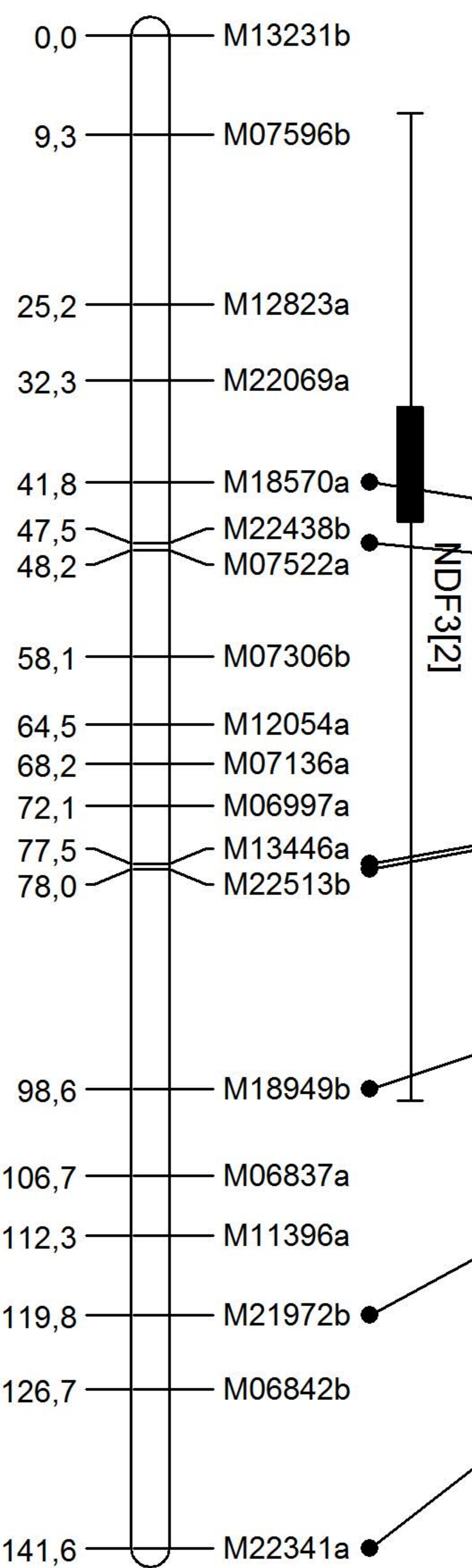

3b

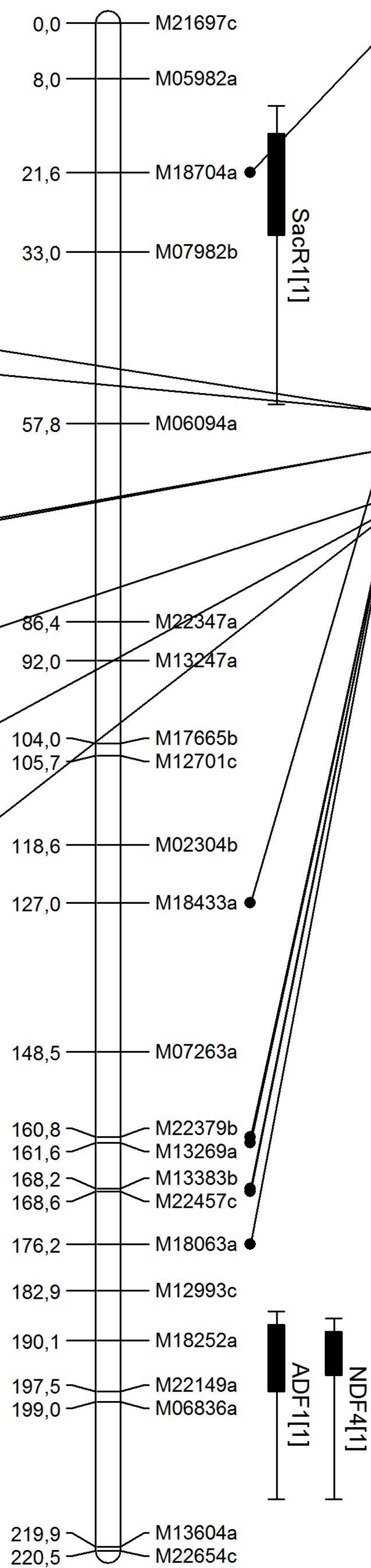

SB03

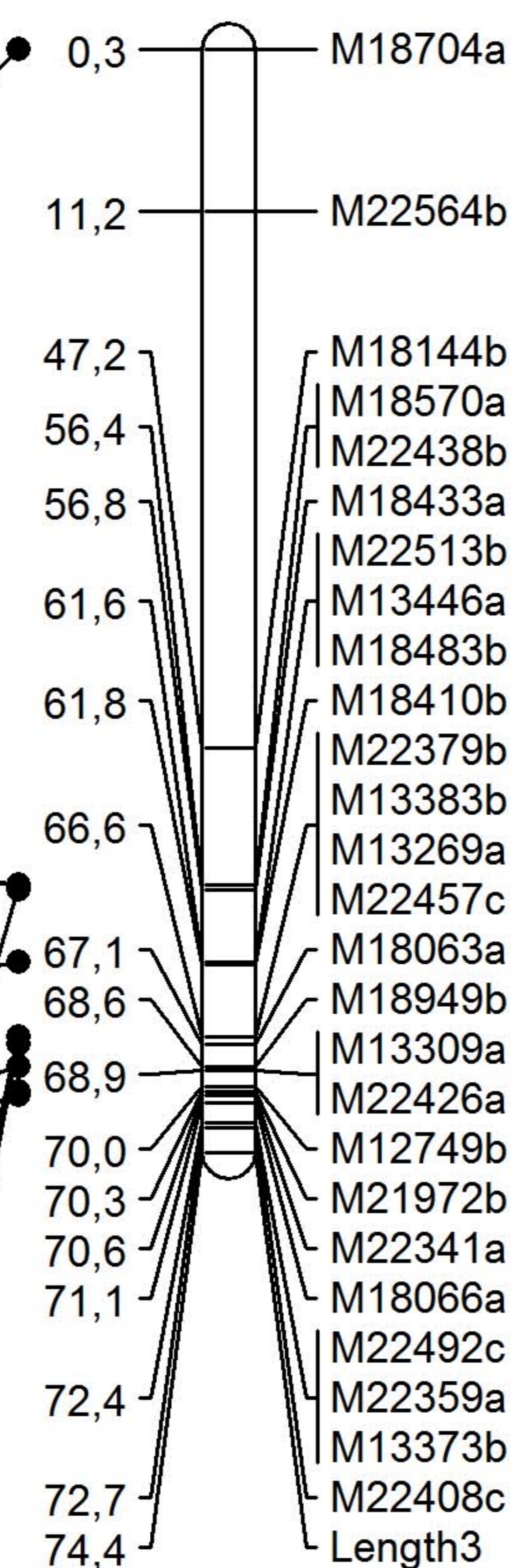

3c

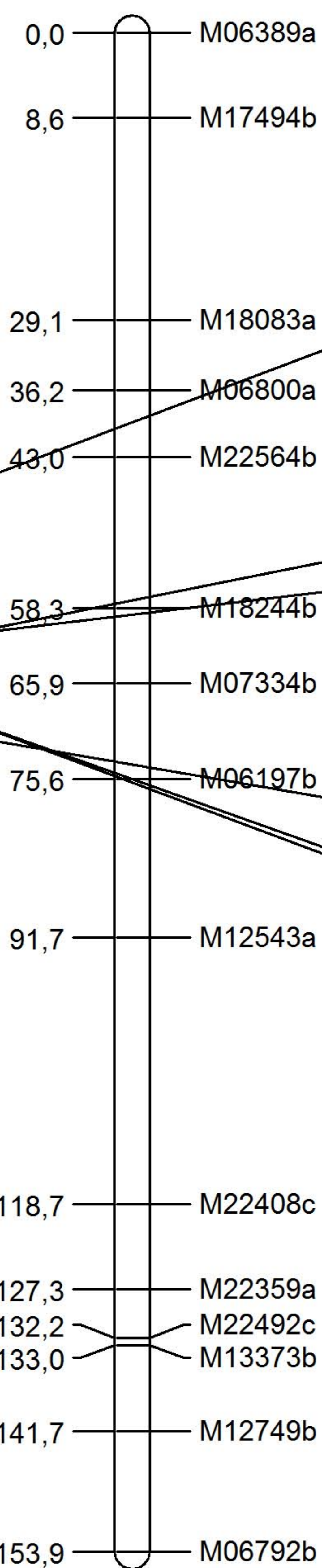

3dR

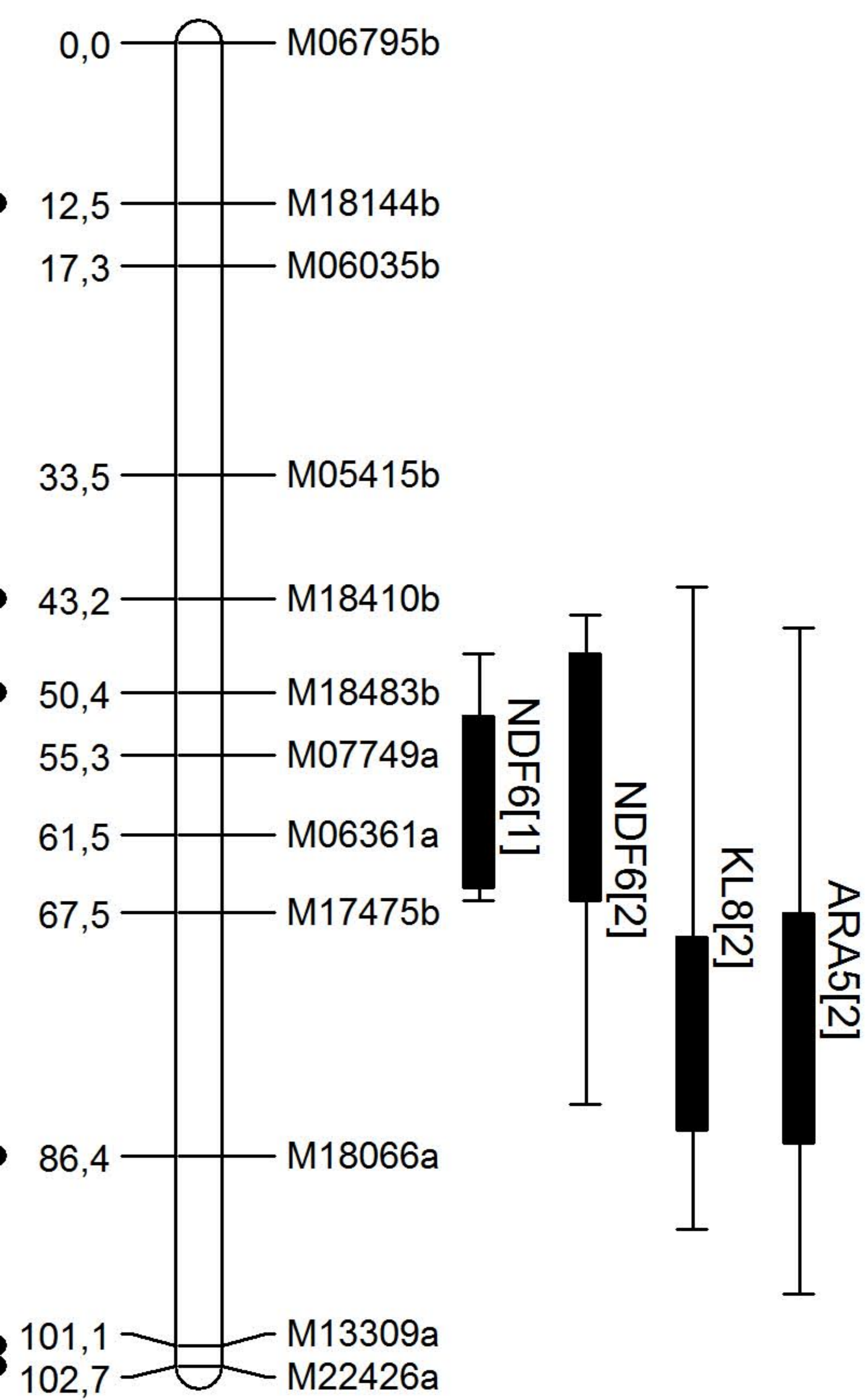

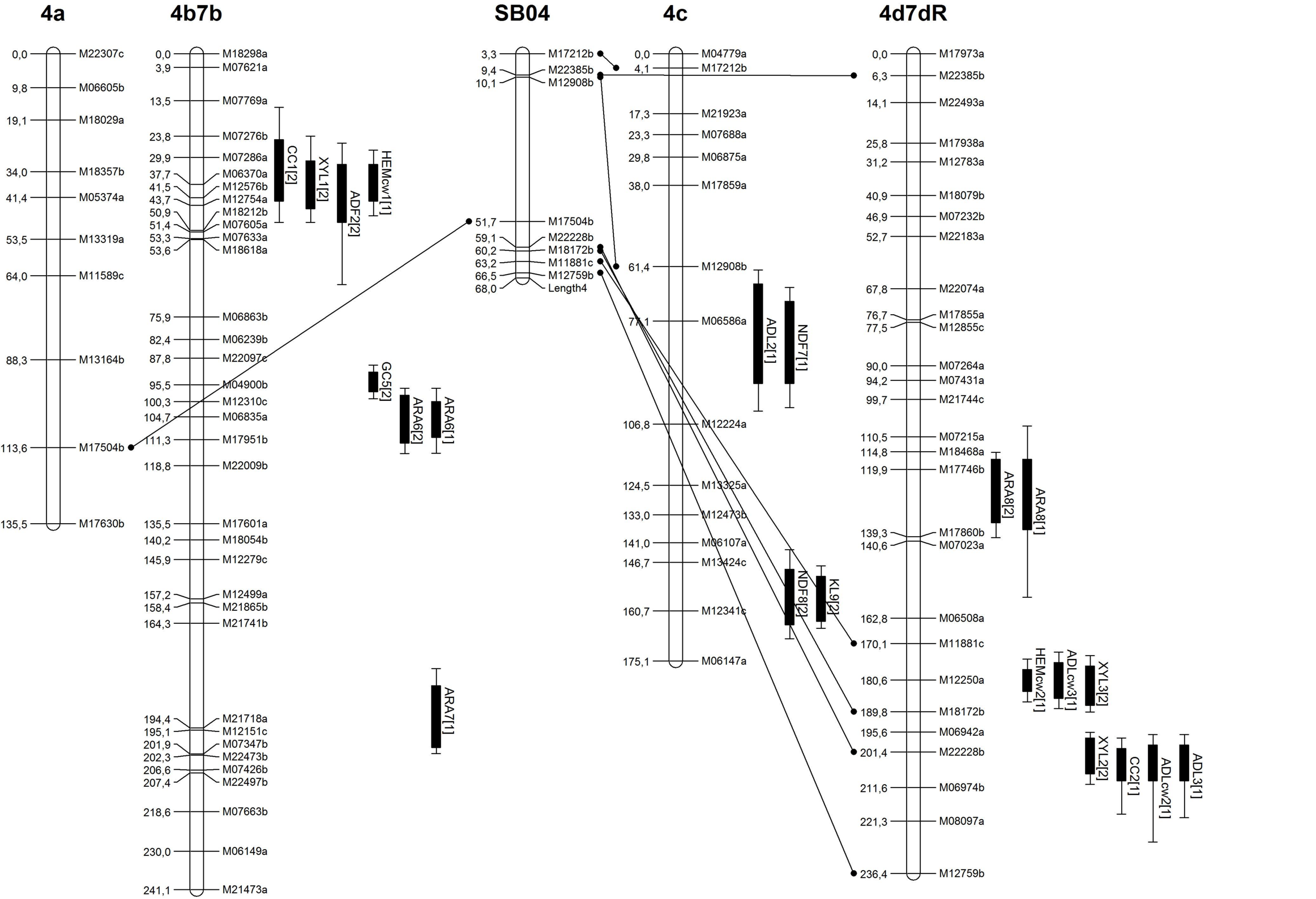

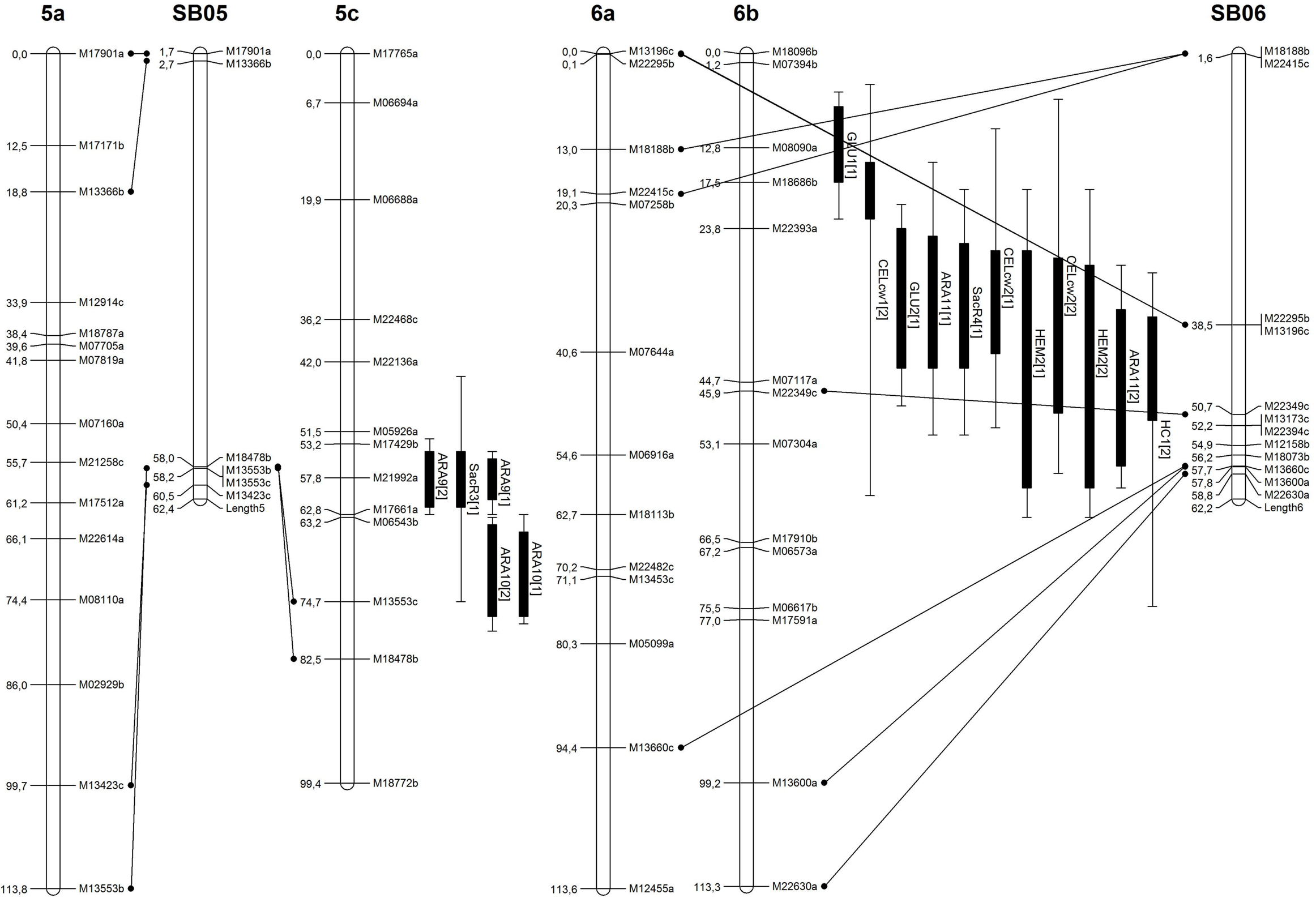

SB06

6c

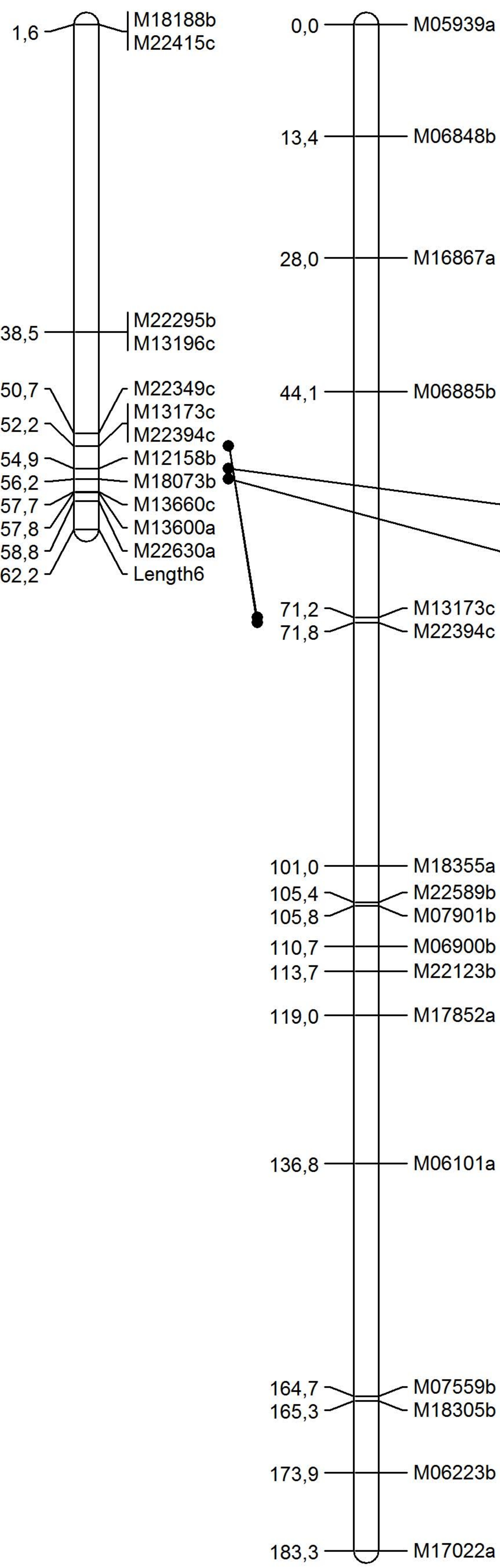

6d

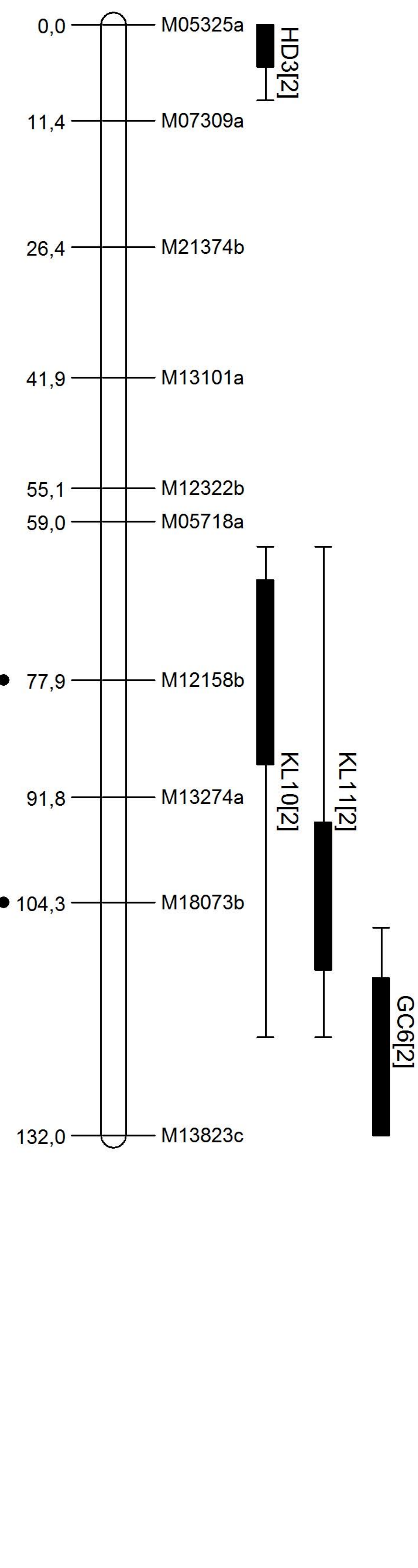

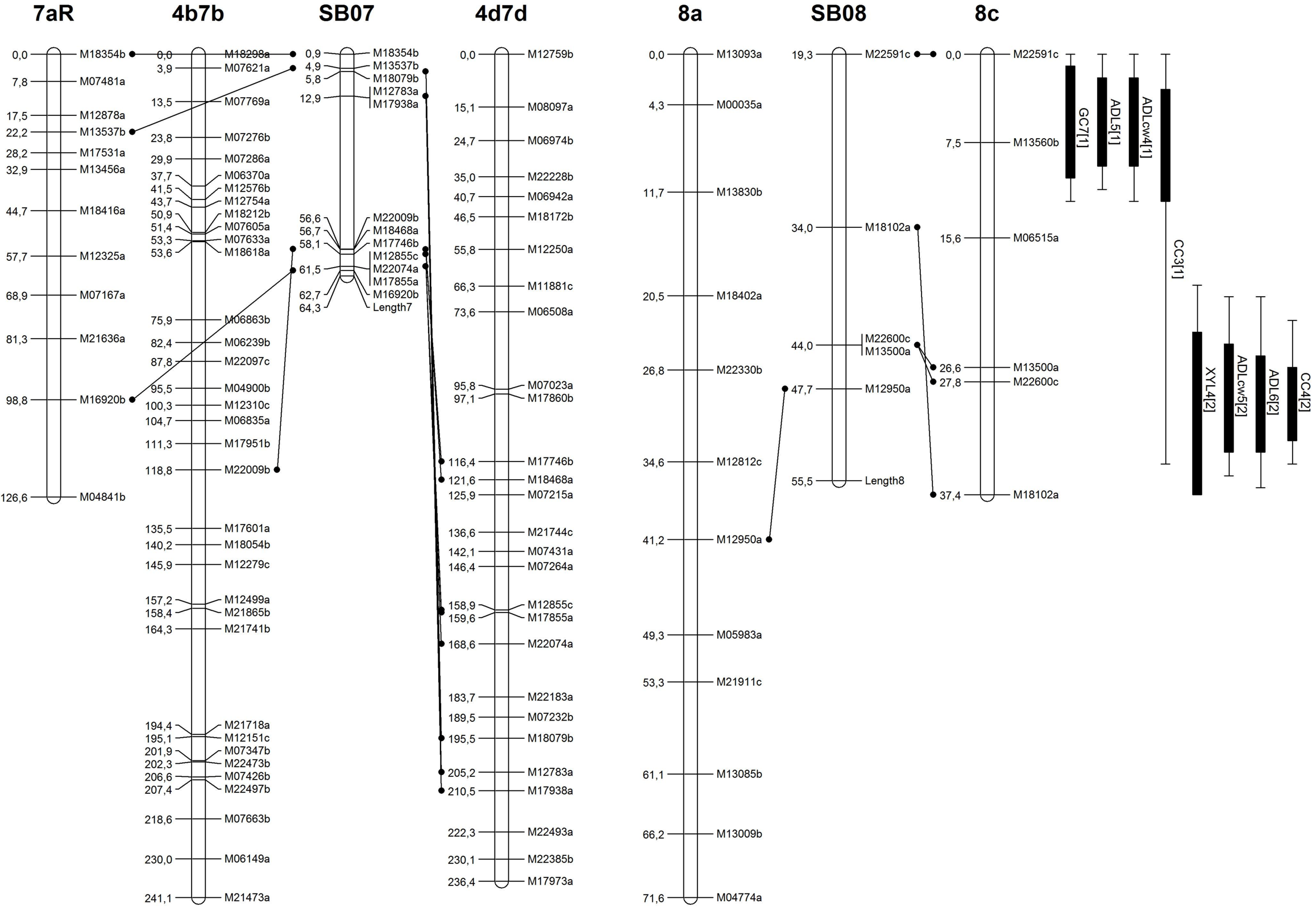

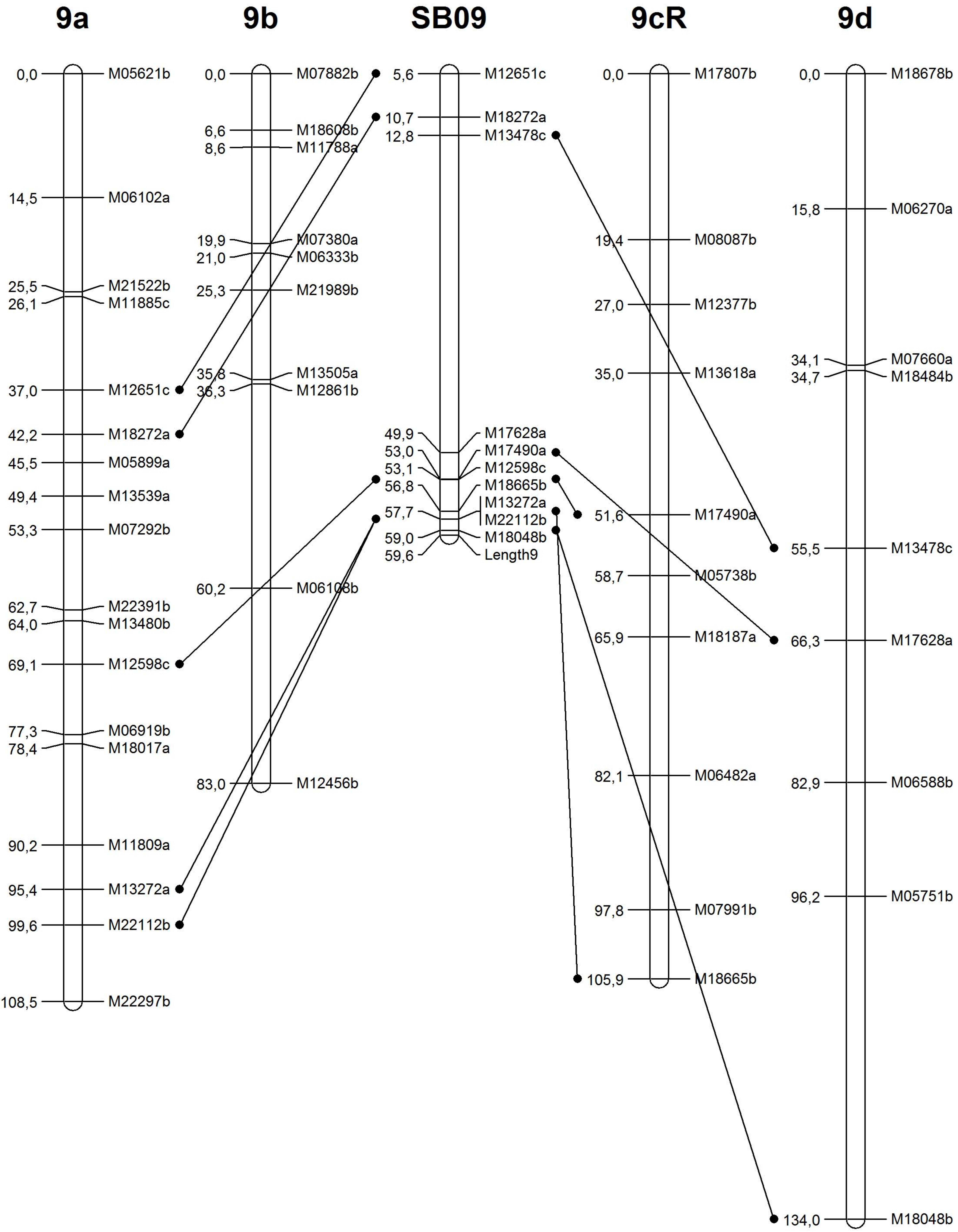

10aR

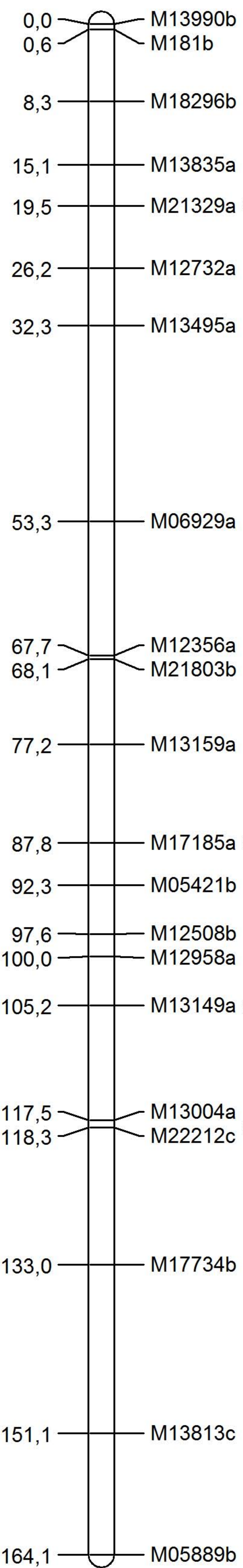

10bR

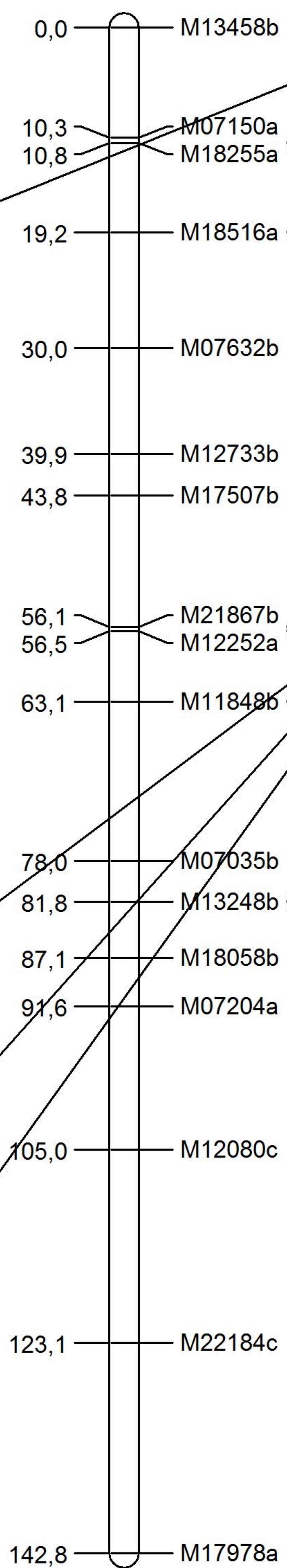

SB10

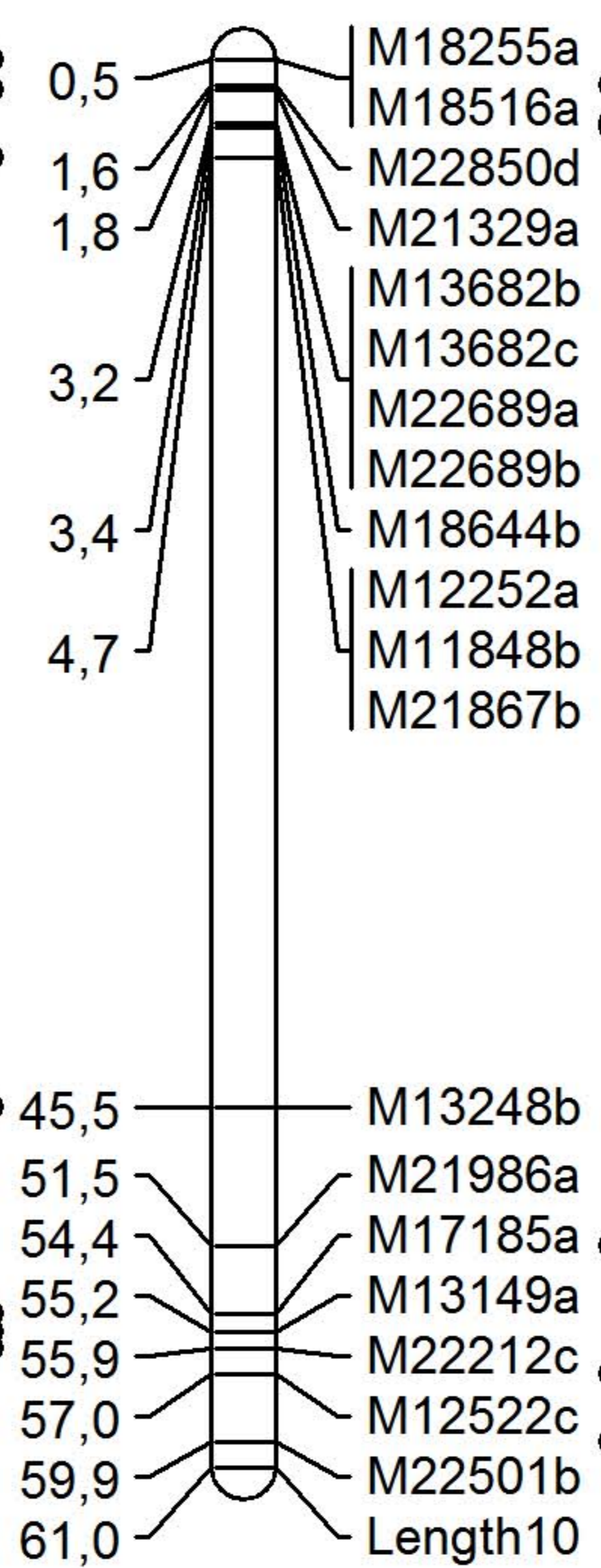

10cR

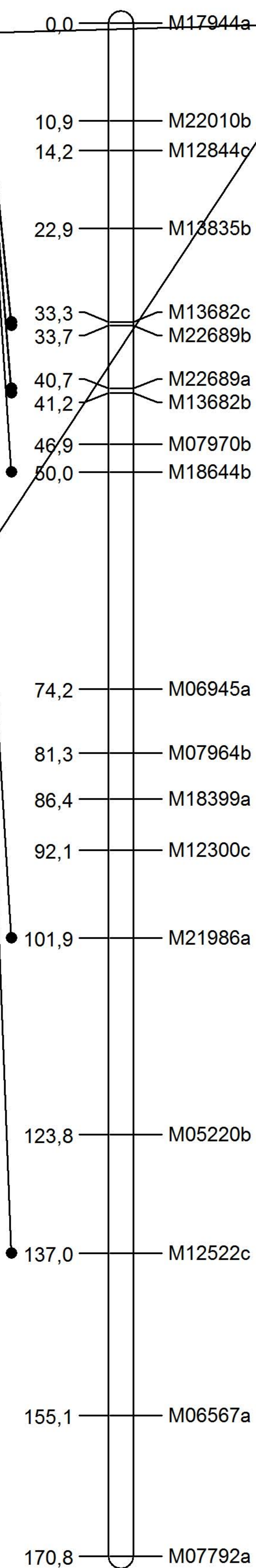

10d

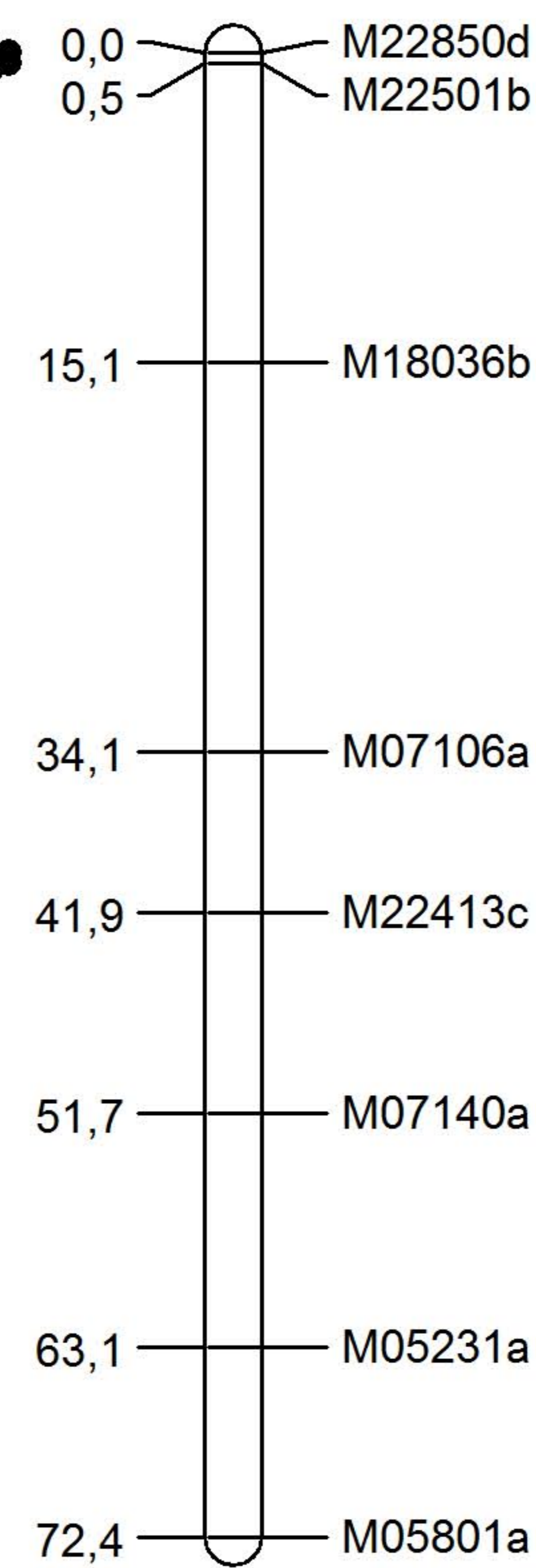

GLU4[1]

SacR7[1]

ARA15[2]

Supplement: Additional file 1: Figure S1. — Synteny map depicting the alignment and localization of M. sinensis mapped markers to the Sorghum bicolor (L.) Moench genome. Linkage groups of the female map are designated as ‘a’ or ‘b; linkage groups of the male map are designated as ‘c’ or ‘d’. The position of mapped QTLs is also shown. For each QTL, colored boxes indicate 1-LOD support intervals while extension bars delimit 2-LOD support intervals. (PDF 5499 kb) [file 12864_2017_3802_MOESM1_ESM.pdf]
